# Gut microbiota and spleen-strengthening and dampness-dispelling therapies in obesity and related metabolic disorders: key mechanisms and therapeutic potential

**DOI:** 10.3389/fphar.2026.1674533

**Published:** 2026-03-11

**Authors:** Huiyi Peng, Jincheng Jiang, Zhoujin Tan, Ping Jiang

**Affiliations:** 1 The First Hospital of Hunan University of Chinese Medicine, Changsha, Hunan, China; 2 School of Traditional Chinese Medicine, Hunan University of Chinese Medicine, Changsha, Hunan, China; 3 The World-class Disciplinary Cultivation of Chinese Medicine in Hunan Province, Changsha, Hunan, China

**Keywords:** energy metabolism, gut microbiota, obesity, potential mechanism, strengthening the spleen and dispelling dampness, traditional Chinese medicine

## Abstract

Obesity, characterized by excessive body fat and systemic low-level inflammation, is closely linked to energy imbalance and gut dysbiosis. Traditional Chinese medicine (TCM) attributes obesity to spleen deficiency and dampness excess, advocating “strengthening the spleen and expelling dampness” as the core treatment strategy. Studies have shown that TCM formulas, botanical drugs, and their active metabolites can significantly enhance the balance of the gut microbiota, correcting lipid and glucose metabolism disorders, and effectively reducing chronic inflammation and oxidative stress. This study summarizes and concludes relevant research, systematically elaborating on the relationships among TCM formulas, botanical drugs, their metabolites for strengthening the spleen and dispelling dampness, and the gut microbiota. Try to elucidate their effects on obesity by regulating the gut microbiota.

## 1 Introduction

Obesity is a persistent metabolic imbalance characterized by excessive adipocytes, uneven fat allocation, and localized fat accumulation (Chinese Medical Association, 2020). It is currently widely believed that, for adults, a body mass index (BMI) ≥ 30 kg/m^2^ indicates obesity, whereas for children, age should be taken into consideration (Chinese Medical Association, 2020; Lingvay et al., 2024; World Health Organization, 2024). It is regulated by diet, age, and other factors, often in combination with other diseases (Fan et al., 2023; Murga-Garrido et al., 2023). Obesity affects 800 million people worldwide, and the World Health Organization (WHO) predicts that it will exceed 2 billion by 2035 (Safaei et al., 2021; Perdomo et al., 2023; Trust for America’s Health, 2024). Obesity poses significant challenges to patients, families, and society and is emerging as a pressing global health concern (World Health Organization, 2024; World Obesity, 2024). Therefore, it is crucial to address obesity management. The gut microbiota has attracted much attention for its potential role in the development and treatment of obesity.

Modern medical research has found that biological disorders caused by a high-calorie diet can lay the foundation for obesity by altering metabolic pathways and gene expression (Qin et al., 2018; Chanda and De, 2024; Leite et al., 2024). The intake of probiotics can effectively reduce fat accumulation and improve blood sugar regulation (Koutnikova et al., 2019; Guo et al., 2024). Its mechanisms involve the dynamic regulation of energy metabolism by the gut, microbiota, and host (such as the brain-gut axis and the gut-microbiota-immune axis) (Boulangé et al., 2016; Asadi et al., 2022). Traditional Chinese medicine (TCM) attributes obesity to spleen deficiency and dampness excess, meaning that the spleen’s function is impaired, unable to transport and transform nutrients, leading to the formation of phlegm-dampness that hinders digestion and absorption. Therefore, TCM emphasizes “strengthening the spleen and expelling dampness” as the main principle for the prevention and treatment of obesity. From this perspective, the TCM and microecology both believe that the core of obesity is an imbalance in energy metabolism. Therefore, we focused on formulas and botanical drugs that can strengthening the spleen and dispelling dampness. Summarizing the main findings and research gaps of these studies can provide new perspectives and ideas for TCM to regulate intestinal microecology and treat diseases.

## 2 Literature search and methods

This study first consulted the digital library of Hunan University of Chinese Medicine and the Duxiu database to identify ancient Chinese texts on formulas that strengthen the spleen and expelling dampness. After summarizing these formulas, only the top 10 high-frequency formulas were retained. The main botanical drugs in these formulas were used to screen for key substances. The key metabolites within these botanical drugs were filtered from the TCMSP database (oral bioavailability ≥30% and drug-likeness ≥0.18). A comprehensive literature search was conducted using PubMed, Web of Science, and CNKI. The main search terms included “traditional Chinese medicine”, “obesity”, “BMI”, “overweight”, “gut microbiota”, “microbiome”, specific formula names (e.g., “Sijunzi Decoction”, “Shenling Baizhu Powder”), and metabolites (such as Glabridin, Isoliquiritigenin, Quercetin, Naringenin). Free searches were also conducted during the retrieval. The publication date of the retrieved literature was cut off at April 2025. Inclusion criteria: Original research (*in vitro*, *in vivo*, and clinical studies) published in English and high-quality reviews were considered first. Reviews needed to involve the relevant mechanisms of gut microbiota and obesity. Exclusion criteria: Included unpublished or retracted articles, as well as articles that did not involve Chinese medicine and its related monomers, and content whose core theme was not directly relevant.

## 3 Obesity and gut microbiota

**FIGURE 1:**
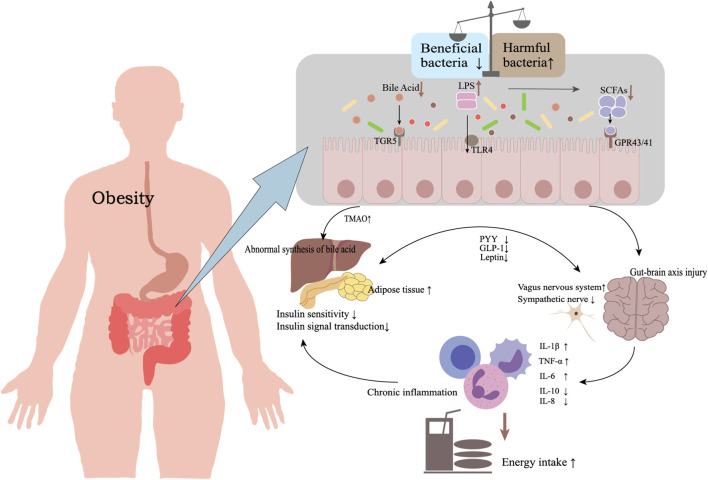
The role of gut microbiota in obesity development. Notes: Obesity is an imbalance of energy metabolism caused by chronic inflammation, brain-gut axis damage, and abnormal bile acid metabolism, and is related to the gut microbiota and its related metabolic products. Abbreviations: TGR5, G protein-coupled bile acid receptor-5; TLR4, toll-like receptor 4; LPS, lipopolysaccharide; SCFAs, short-chain fatty acids; GPR43/41, G protein-coupled receptors 43/41; TMAO, trimethylamine-N-oxide; PYY, peptide YY; GLP-1, glucagon-like peptide 1; TNF-α, tumor necrosis factor-α; IL-6, interleukin-6; IL-10, interleukin-10; IL-8, interleukin-8; IL-1β, interleukin-1β.

The pathological basis of obesity is the increased and dysfunctional adipose tissue. Adipose tissue regulates energy and participates in various pathophysiological processes in the human body, including immune and inflammatory responses and bone metabolism (Khanna et al., 2025). As an important metabolic organ, the gut microbiota interacts dynamically with the host. Changes in external factors such as diet and the environment can cause microbiota dysbiosis, which is closely related to the development of obesity (Gentile and Weir, 2018; Keshet and Segal, 2024; Van Hul et al., 2024). And in recent years, there has been an increasing amount of evidence showing the link between the gut microbiota and obesity. Energy metabolism imbalance directly or indirectly by the gut microbiota and its metabolic products is involved in the occurrence and development of obesity (Figure 1).

### 3.1 Gut microbiota and short-chain fatty acids (SCFAs) on energy metabolism

The research by SATO et al. indicates that an imbalance in the gut microbiota (lower abundance of several *Clostridium* species) and a reduction in SCFAs are associated with the onset of obesity (Sato et al., 2024). Kulkarni et al. found that gut dysbiosis induced obesity (Kulkarni et al., 2023). Also, changes in the expression of lipid metabolism genes. Germ-free (GF) mice could resist obesity on a high-fat diet, likely linked to enhanced fatty acid metabolism (Bäckhed et al., 2007). When the gut microbiota of normal mice was transplanted into GF mice, energy synthesis increased (Bäckhed et al., 2004). All of the above indicate that the gut microbiota participates in regulating energy intake and storage in obesity.

SCFAs are important metabolites produced by the gut microbiota that affect glucose and lipid metabolism. By adjusting the proportions of different SCFAs in the mouse diet, the characteristics of the gut microbiota are altered, and the expression of G protein-coupled receptors 43/41 (GPCR43/41) in intestinal cells changes (Lu et al., 2016). These changes can also affect the release of glucagon-like peptide-1 (GLP-1), peptide YY (PYY), and leptin, adjust insulin sensitivity, and affect glucose metabolism (Samuel et al., 2008; Lu et al., 2016). This mechanism has also been verified in obese patients (Chambers et al., 2015). Some studies have indicated that butyrate and propionate may be involved in adjusting food intake to suppress weight gain, prevent diet-related obesity, and insulin resistance (Lin et al., 2012). This involves the direct inhibition of histone deacetylase (HDAC) by the adenosine 5’‐monophosphate-activated protein kinase (AMPK) pathway, increases fatty acid oxidation, and regulates energy balance (Bäckhed et al., 2007; Hong et al., 2016). All the studies are summarized in Table 1.

**TABLE 1:** Summary of the study of gut microbiota and SCFAs on energy metabolism.

Study	Design	Measurement	Gut microbiota	Metabolites related to microbiota	Associated factors
SATO et al.	sPLA2-X-deficient (Pla2g10−/−) mice	A high-fat diet (60% fat calories)	*Lachnospiraceae NK4A136 group↓, Lachnoclostridium↓, Roseburia and Turicibacter↓*	SCFAs↓	Adipocyte hypertrophy↑
					Th1, Tc1↑
					Alanine aminotransferase (ALT)↑
					Genes related to protection of the epithelial barrier↓
					Inflammatory genes↑
					ω3 PUFAs ↓
Kulkarni et al.	C57BL/6 mice	Antibiotic depletion, Fecal bacteria transplantation (FMT) (from obese patients)	*Colidextribacter↓, Faecalibaculum↓, Lachnospiracea↓, Oscillibacter↓, Phascolarcctobacterium↓, and Rosburia↓*	—	IL-6, MCP-1 and IL-1β↑
					IL-17 and IFNγ↑
					Genes related to lipid uptake and metabolism↑
Fredrik et al.	Fiaf−/−mice	A high-fat diet (irradiated Western diet)	*Bacteroides thetaiotaomicron↑ and Methanobrevibacter smithii↑*	—	triglyceride↑
					Phospho-AMPK↓
					Serum glucose and insulin↓
Fredrik et al.	Rag1−/− mice	Germ-free(GF) colonization,FMT (*Bacteroides thetaiotaomicron* strain VPI-5482)	—	—	Total body fat content↑
					Metabolic rate↓
					Leptin↑
					Liver triglyceride↑
					Acetyl-CoA carboxylase (Acc1), and fatty acid synthase (Fas)↑
Lu et al.	C57BL/6J male mice	A high-fat diet (60% fat calories, mix with SCFAs or not)	*Firmicutes↑, Bacteroidetes↓,* protein bacteria↑, *Actinobacteria↓*, *Candidate_division_TM7↓, Ruminococcaceae↑, Lachnospiraceae↑, Anaerotruncus↑ and Lactobacillus↑*	—	Glucose, triglycerides, cholesterol and insulin↑
					Free fatty acids↓
					IL-1β, IL-6, IL-10 and MCP-1 ↑
					GPR41 and GPR43↑
					GLP-1 and PYY↑
					cpt1a, cpt1c and cpt2↓
					The genes involved in mitochondrial biogenesis↓
Samuel et al.	Gpr41^−/−^ mice	FMT(B. *thetaiotaomicron* strain VPI-5482 and M. *smithii* strain PS)	—	SCFAs↑	GPR41, GPR43, and GPR120 mRNAs↓
					Fat pat weight↑
					Serum leptin↓
					Fas↓
Chambers et al.	Randomized controlled trial	10 g/day inulin-propionate ester	*Bifidobacterium spp↑, Atopobium cluster↑, Bacteroides/Prevotella ↑*	—	GLP-1 and PYY↑
					Subcutaneous adipose tissue↓
					Aspartate transaminase (AST) and ALT↓
Lin et al.	Free fatty acid receptors 3-deficient mice	A high-fat diet with SCFAs	—	—	leptin↓
					insulin↓
					GLP-1, PYY, and amylin↑
					Plasma ghrelin↓
					GIP↑
Hong et al.	C57BL/6 J male mice	A high-fat diet (40% fat calories), sodium butyrate in drinking water	—	—	The epididymal fat mass↓
					Glucose, insulin and leptin↓
					Triglyceride and total cholesterol↓
					AMP, ATP and ADP↑
					Hormone sensitive lipase (HSL) and lipoprotein lipase (LPL) ↑
					The genes involved in mitochondrial thermogenesis↑
					The genes involved in fatty acid β-oxidation↑
					Phospho-AMPK↑
					Histone deacetylase 1(HDAC1)↓

In summary, the gut microbiota and SCFAs mutually influence each other, directly or indirectly participate in regulating energy, and are involved in the development of obesity.

### 3.2 Gut microbiota in host inflammation

Chronic inflammation is also a key mechanism of obesity. Macrophage-mediated inflammatory responses in white adipose tissue (WAT) are closely related to metabolic disorders of fat (Jamar and Pisani, 2023). Certain metabolites of the gut microbiota (such as SCFAs) can promote the production of anti-inflammatory factors, such as interleukin-10 (IL-10), thereby reducing inflammation and improving insulin sensitivity (Macia et al., 2015; Lu et al., 2016). Conversely, gut microbiota dysbiosis and altered metabolites impair WAT function, further exacerbating systemic chronic inflammation (Virtue et al., 2019; Gaudino et al., 2024). Impairment of intestinal barrier function and abnormalities of gut-associated lymphoreticular tissue (GALT) are important pathways through which gut microbiota dysbiosis leads to inflammation. For example, lipopolysaccharide (LPS), a cell wall component of Gram-negative bacteria, is often carried by extracellular vesicles (EVs). When intestinal barrier permeability increases, LPS can easily leak into the bloodstream, a process known as bacterial translocation. LPS that enters the systemic circulation activates immune cells (such as macrophages), inducing the occurrence of systemic chronic low-grade inflammation (Cani et al., 2007). Studies have shown that factors such as a high-fat diet can disrupt the gut microbiota balance, increase the LPS levels (Jain et al., 2024). LPS activates various inflammatory signaling pathways by binding to Toll-like receptor 4 (TLR4), releasing pro-inflammatory cytokines such as tumor necrosis factor-α (TNF-α) and interleukin-6 (IL-6), triggering a systemic inflammatory response (Cani et al., 2007; Kim and Sears, 2010; McKernan et al., 2020). All the studies are summarized in Table 2.

**TABLE 2:** Summary of the study of gut microbiota on response of host inflammation.

Study	Design	Measurement	Gut microbiota	Metabolites related to microbiota	Associated factors
Macia et al.	GPR43^−/−^ and GPR109A^−/−^ mice	High fibre diet, sodium acetate in drinking water	*Bacteroidiaceae*↓, *TM7* and *Oscillibacter* ↓, *Prevotellaceae↑, Lachnospiraceae↑, Alcaligenaceae↑* and *Helicobacteraceae*↑	—	IL-18↑
Gaudino et al.	IL22^−/−^ mice	A high-fat diet	*Oscillibacter*↑	—	Not affect expression of IL-1β
					Ileal lipid metabolism genes↑
Virtue et al.	miR-181-deficient mice (DKO)	A high-fat diet, antibiotic depletion, FMT(*tryptophanase-sufficient* or *tryptophanase-deficient* strains of *E. coli.*)	—	Indole-3-carboxylic acid and indoxyl sulfate↓	Lipid droplet deposition↑
					Lipid catabolizing (Acox1 and Lipe) genes↑
					Glucose clearance↓
					triglycerides↑
					Ppara, Lipe, and Pnpla2(triglyceride hydrolyzer)↓
					Epididymal white adipose tissue↑
Cani et al.	CD14 mutant mice	A high-fat diet (72% fat), infusion of LPS	*Cytophaga*↓*, Eubacterium rectale-Clostridium coccoides group*↓ and *Bifidobacteria*↓	—	O2 consumption, CO2 production ↑
					insulin↓, insulin resistance↑
					Tregs, eosinophils, and ILC2s↑
					M2 macrophage polarization genes↑
					Pro-inflammatory M1 macrophage polarization genes↓
					Fasting blood-glucose↑
Jain et al.	C57BL/6 male mice	A high-fat diet (60% fat calories)	*Proteobacteria*↑	—	Fasted insulin↑
					TNF-α, IL-1, IL-6, and PAI-1 mRNA↑
					LPS on the surface of faecal-EVtotal↑
Mckernan et al.	TLR4−/− male mice	A diet with 4.5% fat, mouse bone marrow cells were cultured (LPS or palmitate treatment)	—	—	TLR4↑
					F4/80, CD86 and CD206↑
					Inflammatory responses genes↑
					lipid↑
					Pparγ, cebpα, and Pgc1α(genes)↓
					TNF-α,IL-6 and MCP1↑

Gut microbiota dysbiosis, inflammation, and obesity are interlinked in a vicious cycle.

### 3.3 Gut microbiota participates in bile acid metabolism

Gut microbiota dysbiosis is a key factor in abnormal bile acid (BA) metabolism and obesity. Obese patients often have gut microbiota dysbiosis, with BA metabolism disrupted, as evidenced by abnormal portal vein bile acids (BAs) (Aydin et al., 2025). The activity of key enzymes (such as cholesterol 7α-hydroxylase (CYP7A1)) and receptors (such as farnesoid X receptor (FXR)) involved in bile acid synthesis can influence energy expenditure, a process associated with changes in the gut microbiota (Sayin et al., 2013; Wahlström et al., 2016; Parséus et al., 2017; Zheng et al., 2017). Wang et al. also found that after fecal microbiota transplantation, the BAs in mice changed, affecting GLP-1 levels through the G protein-coupled bile acid receptor-5 (TGR5) pathway, thereby promoting fat metabolism and energy expenditure, which has a positive effect on preventing obesity (Wang et al., 2023). Dysbiosis of the gut microbiota is accompanied by an increased production of trimethylamine-N-oxide (TMAO), which interferes with the binding of bile acids to FXR and promotes fat synthesis, exacerbating obesity (Tan et al., 2019; Mo et al., 2025). All the studies are summarized in Table 3. In conclusion, the status of the gut microbiota can affect bile acid metabolism, energy metabolism balance, involved in obesity.

**TABLE 3:** Summary of the study of gut microbiota participates in bile acid metabolism.

Study	Design	Measurement	Gut microbiota	Metabolites related to microbiota	Associated factors
Adyin et al.	Cohort study	The ages of ≥18 and ≤64 years Body mass index of ≥35 kg/m2	*Actinomycetota*↑, *Bacillota*↑, *Bacteroidota*↑ and *Pseudomonadota*↑	BAs profiles in portal↑	FGF19 in portal↑
				Diversity of BAs in portal↓(Glycocholic acid (GCA), glycodeoxycholic acid (GCDCA), glycerodeoxycholic acid (GDCA), taurocholic acid (TCA), taurochenodeoxycholic acid (TCDCA), taurodeoxycholic acid (TDCA))	
Sayin et al.	FXR^−/−^ mice	Antibiotic depletion, GF feeding, TCA or a mixture of TCA and (tauro-β-muricholic acid)TβMCA	—	BAs↑, taurine↑	Cholesterol and phospholipids↓(GF mice)
				TβMCA↑, TCA and Tauro-α-muricholic acid (TαMCA)↓	CYP7A1↑(GF mice)
					Bile acid transporters genes↑(GF mice)
					Molecular targets Shp and Fgf15↓
					FXR↓
Ava et al.	FXR^−/−^ germ-free mice	GF feeding, FMT (from FXR^−/−^ mice with high-diet)	*Bacteroidia*↓ *Firmicutes*↑	—	Fasting glucose↑
					Oral glucose tolerance↓
					Macrophage markers Emr1 (encodes the protein F4/80)↑
					Saa3 and Tnfa (encode proinflammatory cytokines) ↑
					Ccl2 (promotes macrophage infiltration into WAT)↑
					Triglycerides, saturated triglycerides and cholesteryl esters↑
					Fatty acid transporter Cd36, Apoc2 and Vldlr↓
Zheng et al.	C57BL/6 male mice	A high-fat diet supplemented with GW4064 (FXR agonist)	*Firmicutes*↑, *Proteobacteria*↑, and *Actinobacteria*↑; *Verrucomicrobia*↓, *Bacteroidetes*↓ and *TM7*↓; *Ruminococcus gnavus*↑, *Blautia* spp.↑, *Oscillospira spp*.↑, and *Bilophila* spp.↑	Total BAs, cholic acid(CA),GCA,TDCA,TCA, chenodeoxycholic acid (CDCA), deoxycholic acid (DCA),TCDCA, β-muricholic acid (βMCA), lithocholic acid (LCA), and 7-ketolithocholic acid (7-ketoLCA)↑, β-cholic acid (βCA)↓	Bady weight↑
Wang et al.	Gpbar1 (known as TGR5) global knock out(KO) male mice	Antibiotic depletion,GF colonization(C57BL/6J donor mice)	—	Total BAs↑, TCA and TβMCA↓	GLP-1↓
					TGR5↓
Mo et al.	SD male rat	A high-fat diet (45% fat calories), TMAO in drinking water	*Firmicutes*↑, *Mucispirllum*↑; *Bacteroidetes*↓, *Flavonifractor*↓ and *Intestinimonas*↓	SCFAs↓	Muscles mass↓
					lipid↑
					Muc-2 and goblet cells↓
Tan et al.	C57BL/6J male mice	A high-fat diet (45% fat calories), TMAO in drinking water	—	TCA↑, DCA and TDCA↓	AST↑
					Total cholesterol↑
					Hepatic lipogenic genes Srebp-1c and Fas↑
					Medium-chain acyl-CoA dehydrogenase (Mcad) mRNA↓
					Microsomal triglyceride transport protein (Mtp) mRNA↓
					CYP7A1 mRNA↑
					FXR mRNA↑, FXR↓

### 3.4 Gut microbiota involved in the gut-brain axis

The gut-brain axis (GBA) is a complex network connecting the gut, gut microbiota, and their metabolic products with the nervous, immune, and endocrine systems. SCFAs and gut microbiota are associated with the transmission of signals to the central nervous system, regulating energy metabolism and appetite (Lin et al., 2012; Frost et al., 2014; Liu X. et al., 2021). They are also involved in activating the GPR43/GLP-1 pathway, promoting the secretion of GLP-1 and PYY, and improving glucose and lipid metabolism (Samuel et al., 2008; Lu et al., 2016). A high-fat diet can increase gastric inhibitory polypeptide (GIP) levels in the brain and induce leptin resistance through the GIP receptor/Rap1 signaling pathway (Kaneko et al., 2019). Current research has demonstrated that a high-fat diet causes gut microbiota dysbiosis, but whether it is connected to the GIP signaling in the brain remains to be studied. However, gut-brain peptides (such as GLP-1 and PYY) are key to GBA intercommunication. Their expression levels change, and the gut microbiota also changes, both of which participate in obesity. In summary, the gut-brain axis is also a major link in obesity. All the studies are summarized in Table 4.

**TABLE 4:** Summary of the study of gut microbiota involved in the gut-brain axis.

Study	Design	Measurement	Gut microbiota	Metabolites related to microbiota	Associated factors
Liu et al.	Female C57BL/6J mice and its offspring	A high-fat diet	*S24-7*↓*, Bifidobacterium animalis*↓*, Prevotella*↓, and *Clostridiales*↓	Acetate and propionate↓	Three-chamber sociability test, the Y-maze test, PSD-95, FXR1, FXR2, TDP2, GluN2B, and GluA2↓; MAFB, BDNF, NGF, DAP12, and CX3CL1↓, CD31 and F4/1↑
Frost et al.	C57BL/6J male mice	A high-fat diet (41.8% energy from fat), supplemented with inulin or cellulose	—	Total SCFA, acetate↑	Signal intensity in the arcuate nucleus↑, AMPK↓, γ-amino butyric acid (GABA), lactate(Lac), glutamate(Glu)↑
Kaneko et al.	C57BL/6J mice	A high-fat diet (60% calories fat), Gipg013 (GIP receptor antagonist) brain injection	—	—	Glucose, leptin and insulin↓, STAT3↓, GIP ↑, Rasrelatedprotein 1 (Rap1)↑

## 4 TCM-based interpretation of obesity

TCM classifies obesity into categories, such as *fei ren* (fat from top to bottom), *fei man* (abdominal obesity), and *gao ren* (fat in the abdomen and thighs). *Su Wen (Plain Questions)* stated that obesity is caused by eating refined and fatty foods. These foods are indigestible, which inevitably affects qi transport function in the spleen. Therefore, TCM is associated with impaired spleen function in obese individuals.

**FIGURE 2:**
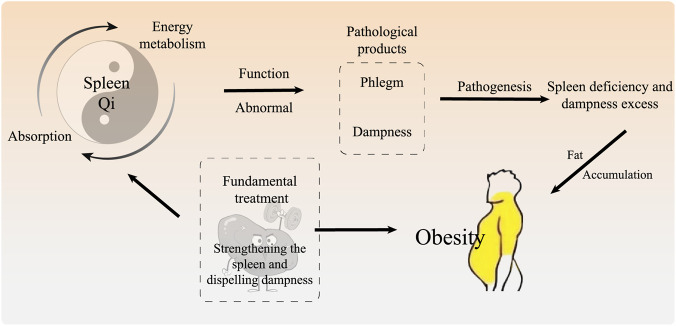
The Occurrence of Obesity under the Understanding of TCM. Notes: TCM believes that spleen deficiency and dampness excess caused by deficiency of spleen qi is the key to obesity. Phlegm and dampness are the pathological product, which in turn affects the function of the spleen. Therefore, the principle of treatment is to strengthening the spleen and dispelling dampness.

TCM holds that the spleen is an important visceral system responsible for the absorption of food and the balance of energy metabolism. Its core function is “transportation and transformation,” that is, transforming food into “subtle substances” that nourish the entire body and maintain the normal metabolism of body fluids. Damage to the spleen’s transportation and transformation function will lead to disorders in fluid metabolism, abnormal energy conversion, and consequently produce “phlegm-dampness.” Phlegm-dampness is both a pathological product and a pathogenic factor that can obstruct the flow of Qi, further affecting the spleen’s transportation and transformation function. Phlegm and dampness stop accumulating in the body, leading to obesity. Therefore, TCM identifies “spleen deficiency and dampness excess” as the primary causes of obesity, with “strengthening the spleen and dispelling dampness” as the fundamental treatment approach. “Strengthening the spleen” means replenishing the insufficient or weak spleen qi. This helps restore the normal transportation and transformation of spleen qi. “Removing dampness” is to remove the phlegm/dampness that has been produced. This helps restore smooth qi movement. This can normalize spleen absorption to balance the energy metabolism (Figure 2).

In simple terms, in the context of obesity, spleen deficiency in Traditional Chinese Medicine is similar to abnormalities in gastrointestinal function and immune function. Dampness excess usually similar to changes in substances with a certain fluidity, such as fluids and blood lipids, or the occurrence of chronic inflammation. Therefore, spleen deficiency often implies changes in gastrointestinal motility (such as gastric emptying rate), gastrointestinal hormones (such as gastrin), and immune indicators (such as CD4/CD8), while dampness excess often implies changes in blood lipids, cholesterol, and inflammatory factors, such as interleukin-2 (IL-2), interferon-gamma (IFN-γ), and reactive oxygen species (ROS). To better understand TCM concepts, we have also classified the indicators from the following literature into two categories: Pi deficiency and Dampness excess (Table 5).

**TABLE 5:** Comparison table of TCM concepts and indicators.

TCM concepts	Biomedical manifestations
Spleen Qi deficiency	Gastric (GAS)
	Motilin (MTL)
	Somatostatin (SS)
	CD4/CD8
	ATP
	AMPK
	MMP
	Gastric emptying
	Small intestinal propulsion
	5-HT
	Visceral sensitivity
	Treg cells
	D-xylose content
	IFN-γ
Dampness excess	IL-2
	T-CHO
	ROS
	IL-6
	TNF-α
	IL-17
	IL-10
	TG
	HDL
	LDL
	SOD
	MDA

## 5 The potential mechanism of TCM formulas in treating obesity

### 5.1 Sijunzi decoction

The Sijunzi decoction was initially documented in the *Taiping Huimin Heji Bureau Prescription,* authored by a Song Dynasty official. This traditional formula, which includes ginseng (*Panax ginseng* C.A.Mey. raw dried root), Atractylodes macrocephala Koidz (*Atractylodes macrocephala* Koidz. raw dried root), Poria cocos (*Poria cocos* (Schw.) Wolf raw dried sclerotium), and Glycyrrhiza (*Glycyrrhiza glabra* L. honey-prepared root and rhizome), serves as a cornerstone remedy for the spleen deficiency syndrome. Sijunzi decoction can participate in energy metabolism, improve inflammatory reactions, and stabilize the intestinal barrier. This process may serve as a viable strategy for combating obesity.

The purified homogeneous polysaccharide from the Sijunzi Decoction, S-3-1, has been shown to modulate essential gut microbiota, including *Lactobacillus*, *Pediococcus*, *Streptococcus*, *Bacteroides*, *Enterococcus*, *Clostridium*, and *Dorea*. Not only that, but also found that acetic acid increased, while Propionic acid and Butyric acid decreased (Gao et al., 2018). Additionally, the non-polysaccharide metabolites of Sijunzi Decoction have been shown to influence gastrointestinal hormone secretion in rats with spleen deficiency, thereby improving gastrointestinal motility. The active polysaccharide metabolites further contribute to repairing the intestinal barrier and bolster immune function by boosting T-lymphocyte activity. During this process, correlation analysis revealed that the levels of inflammatory factors and gastrointestinal (GI) hormones were correlated with changes in the gut microbiota abundance of *Lactobacillus*, *Butyricimonas*, and *Collinsella* (Ma et al., 2021). In addition, Sijunzi decoction inhibited mitochondrial autophagy and restored limb muscle strength in rats with spleen-qi deficiency syndrome by regulating the AMPK/ULK1 signaling pathway (Liu W. et al., 2021).

Liujunzi decoction is a modified formula based on Sijunzi decoction (Pericarpium Citri Reticulatae (*Citrus reticulata* Blanco raw dried pericarp) and Pinellia ternata (*Pinellia ternata* (Thunb.)Breit. ginger-processed rhizome) are added), and its effect on eliminating dampness is stronger than that of the Sijunzi decoction. Clinically, drugs can be added based on the Liujunzi decoction. Research indicates that the Chaishao Liujunzi Decoction (Based on Liujunzi decoction, Bupleurum chinense (*Bupleurum chinense* DC. raw dried root and rhizome), and *Paeonia lactiflora* Pall (raw dried root)) significantly boosts the levels of beneficial gut bacteria, including *Faecalibacterium* and *Fusicatenibacter,* while curbing the growth of harmful strains such as *Erysipelotrichia* (Xing et al., 2024). Another modified prescription of Liujunzi Decoction, Xiangsha Liujunzi Decoction (Based on Liujunzi decoction, *Aucklandia lappa* Decne. raw dried root and *Amomum villosum* Lour. raw dried ripened fruit are added), can improve gastrointestinal motility disorders due to spleen deficiency by inhibiting mitochondrial autophagy and division mediated by PINK1/Parkin (Zhang J. et al., 2022) and can reduce intestinal 5-Hydroxytryptamine (5-HT) signaling by lowering tryptophan hydroxylase-1 (TPH1) expression levels, thereby alleviating visceral hypersensitivity (Zhao et al., 2020). Lin et al. found that Xiangsha Liujunzi Decoction can reduce gastrointestinal inflammation by inhibiting tumor necrosis factor-alpha (TNF-α) and IL-6 *via* downregulation of TLR4, mitogen-activated protein kinase (MAPK), nuclear factor kappa-B (NF-κB), and nitric oxide (NO) levels (Lin et al., 2016).

In summary, the essential mechanism by which Sijunzi Decoction improves spleen deficiency is through regulating energy, restoring gastrointestinal function, and alleviating inflammation, throughout which the gut microbiota also changes.

### 5.2 Shenling Baizhu powder

Shenling Baizhu Powder is composed of ginseng, Atractylodes macrocephala Koidz, Chinese yam (*Dioscorea opposita* Thunb. raw dried root and rhizome), Poria cocos, Glycyrrhiza, *Nelumbo nucifera* Gaertn. raw dried mature seed, *Dolichos lablab* L. raw dried mature seed, *Coix lacryma-jobi* L. var. *mayuen *(Roman.) Stapf raw dried mature seed, *A. villosum* Lour. raw dried ripened fruit, *Platycodon grandiflorum *(Jacq.) A. DC. raw dried root. Shenling Baizhu powder helps lower serum TH1 cytokine levels in ulcerative colitis patients with bloody purulent stool, as evidenced by reduced IL-2 and IFN-γ levels, and alleviates clinical symptoms (Li S. et al., 2021). It can also reduce the intestinal inflammation of rotavirus enteritis mediated by inflammatory factors such as interleukin-1β (IL-1β), IL-6, through the TLR4/Myeloid Differentiation Primary Response Gene 88 (MyD88)/NF-κB pathway (Wang et al., 2021), Zhang et al. also discovered this mechanism in a high-fat diet rat model, there is a reduction of the NOD-like receptor family, pyrin domain containing 3 inflammasome (NLRP3) and caspase 1 (Zhang et al., 2018). Xiao et al. also found that Shenling Baizhu Powder can regulate the intestinal absorption function of rats with spleen qi deficiency, modulate Treg cell-related immunity, decrease IL-17, and increase IL-10 (Xiao et al., 2021). Therefore, Shenling Baizhu Powder can modulate inflammation and immune responses.

Some studies also found that Shenling Baizhu Power can regulate the gut microbiota. Qiao et al. found that Shenling Baizhu San can reduce the levels of low-density lipoprotein (LDL), malondialdehyde (MDA), IL-17, and IL-6 caused by lard in mice. This means that Shenling Baizhu San can improve lipid metabolism and alleviate oxidative stress. In this process, *Lactobacillus* and SCFA levels increased, which are correlated with the aforementioned indicators (Qiao et al., 2024). Lai et al. found that Shenling Baizhu Powder can reduce opportunistic bacteria, increase *Bacilli* and *Lactobacillales*, regulate BA metabolism, increase cyclic AMP (cAMP) levels, and improve intestinal damage and diarrhea caused by drug toxicity (Lai et al., 2022). Meanwhile, Zhang et al. found that Shenling Baizhu Powder not only can improve inflammation and fat metabolism, but also can increase the *Bacteroidetes*/*Firmicutes* ratio and *Bifidobacterium*, reduce *Akkermansia* (Zhang et al., 2018). Zhang et al. found in a spontaneous obese type 2 diabetic rat model that Shenling Baizhu Powder can improve lipid metabolism and blood glucose levels, decrease *Firmicutes* (such as *Lactobacillus*), and increase *Bacteroidetes* (such as *Prevotella*) (Zhang L. et al., 2022).

In summary, Shenling Baizhu Powder can indeed improve lipid metabolism, alleviate inflammation, and regulate the gut microbiota.

### 5.3 Erchen decoction

In TCM, it is believed that a high-fat diet can weaken the spleen and lead to the accumulation of excess moisture in the body. Erchen decoction is often used to treat obesity and hyperlipidemia, and constitutes a fundamental clinical approach for treating damp phlegm syndrome. The erchen decoction consists of Pinellia ternata, Pericarpium Citri Reticulatae, Poria cocos, and Glycyrrhiza.

Zhang et al. found that Erchen decoction can reduce the fat content in mice fed a high-fat diet by increasing peroxisome proliferator-activated receptor γ (PPARγ) and lipoprotein lipase (LPL) (Zhang et al., 2020). Erchen Decoctioncan also participate in the regulation of lipid metabolism in Zucker Diabetic Fatty Rats through the Insulin receptor substrate 1/protein kinase B/protein kinase A/hormone-sensitive lipase signaling pathway (IRS1/AKT/PKA/HSL signaling pathway) and this pathway is closely related to the levels of *Prevotella*, *Ruminococcus*, *Blautia*, *Holdemania* (Zhao et al., 2021). Zhang et al. not only found that Erchen Decoction can regulate fat metabolism and gut microbiota (such as Upregulate *Coprobacillus* and downregulate *Eubacterium*), but it can also increase butyric acid. This mechanism is regulated through the histone deacetylase 1/histone H3 lysine 9 acetylation pathway (HDAC1/H3K9ac pathway) (Zhang L. et al., 2023). In addition to regulating fat metabolism, Liu et al. found that Erchen Decoction can also reduce levels of inflammatory factors (such as TNF-α and IL-1β) and enhance the mucosal barrier (e.g., by increasing ZO-1 levels). In this process, there was an increase in *Firmicutes* and *Proteobacteria*, a decrease in *Bacteroidetes*, *Cyanobacteria*, and *Verrucomicrobia*, and alterations in SCFAs levels (Liu H. et al., 2021). In addition, Miao et al. also found that Ercheng Tang can increase SOD and glutathione peroxidase (GSH-Px), decrease MDA, while the gut microbiota also changes (such as an increase in the *Bacteroidetes*/*Firmicutes* ratio, *Lactobacillus* and *Akkermansia*, a decrease in *Desulfovibrio*) (Miao et al., 2022).

In summary, Erchen Decoction can help improve gut microbiota dysregulation, regulate fat metabolism, and reduce inflammatory levels. Moreover, changes in gut microbiota are closely linked to the regulation of fat metabolism and inflammatory responses.

### 5.4 Daotan decoction

Obesity can cause polycystic ovary syndrome (PCOS), which is a common cause of female menstrual irregularities, and is usually treated with Daotang Decotion. The Daotan Decoction consisted of Pinellia ternata, Pericarpium Citri Reticulatae, Poria cocos, Glycyrrhiza, Fructus Aurantii Immaturus, and Arisaema heterophyllum Blume.

Cangfu Daotan Decoction is modified from Daotan Decoction, and has the effect of resolving phlegm and transforming dampness. Yi et al. found that it can regulate hormone levels (Decrease testosterone (T) and luteinizing hormone (LH), increase follicle-stimulating hormone (FSH) and estradiol (E2)) and lipid metabolism in obese PCOS model rats, and decrease inflammatory factors (such as IL-2, IL-6 and TNF-α). This is associated with the increased expression of organic anion transporting polypeptides-3A1 (OATP3A1) and organic anion transporting polypeptides-2B1 (OATP2B1) (Yi et al., 2021). Another modified formula of Daotan Decoction, Huanglian Wendan Decoction, can also improve obesity. It can reduce IL-1β and IL-18 expression through the NF-κB/NLRP3 pathway, lower blood glucose levels, and improve insulin sensitivity and insulin resistance (Dong et al., 2021). The research of Li et al. also drew similar conclusions. They found that Huanglian Wendan Decoction can regulate lipid and glucose metabolism in diabetic encephalopathy rats, reduce TNF-α, IL-6, IL-1β, and ameliorate brain injury (Li et al., 2016).

Daotan Decoction has been shown to improve inflammatory responses, insulin resistance, and regulate lipid metabolism, but the mechanisms underlying these effects and their relationship with the gut microbiota remain unclear.

TCM formulas participates in the regulation of gut microbiota and its related mechanisms are shown in the Tables 6, 7.

**TABLE 6:** Summary of pre-clinical studies of TCM formulas.

Formulas	Intervention	Model	Dosages	Target/factors	Mechanism	Gut microbiota	Literature
Sijunzi decoction	S-3-1(purified from Sijunzi decoction)	In vitro	2 mg/mL, 1 mg/mL	Acetic acid↑, propionic acid↓, butyric acid↓	Regulation of gut microbiota and SCFAs	*Bacteroidetes*↑, *Firmicutes*↓, *Oscillospira*↑, *Streptococcus*↓, *Enterococcus*↓, *Clostridium*↑, *Bacteroides*↓, *Dorea*↑, *Lactobacillus*↓, *Pediococcus*↓	Gao et al. (2018)
	Standardized Sijunzi decoction extract	Spleen deficiency syndrome rats, antibiotic depletion	10 g/kg·d, 7 days	Gastrin (GAS)↓, motilin (MTL)↓, somatostatin (SS)↓, Ghrelin (GRHL)↓, interleukin-2 (IL-2)↓, interferon-gamma (IFN-γ)↓, CD4/CD8 ratio↑	The regulation of GI hormones, immunomodulatory effect, modulation of the gut microbiota composition	*Actinobacteria*↑, *Rothia*↑, *SMB53*↑, *Lactobacillus*↓, *Collinsella*↑, *Butyricimonas*↓	Ma et al. (2021)
	Sijunzi decoction	Spleen Qi deficiency rats	1.4 g/d, 2.8 g/d, 5.6 g/d, 14 days	ATP↑, MMP↑, reactive oxygen species (ROS)↓, microtubule-associated protein light chain 3 (LC3)↑, sequestosome 1 (p62)↓, AMPK↓, unc-51 like autophagy activating kinase 1 (ULK1)↓	Inhibition of mitochondrial autophagy	—	Liu et al. (2021)
	Xiangsha Liujunzi decoction concentrated dry powder	In vitro, functional dyspepsia rats	0.36 g/mL, 0.18 g/mL, 0.09 g/mL, 14 days	Gastric emptying↑, small intestinal propulsion↑, autophagosomes↓, LC3↑, p62↓, PINK1↓, mitochondrial reactive oxygen species(mtROS)↓	Improvement of mitochondrial function	—	Zhang J. et al. (2022)
	Xiangsha Liujunzi decoction	Functional dyspepsia rats	1 mL/100 g, 7 days	Incremental balloon pressure↓, number of EC cells↓, paired box gene 4 (PAX4)↑, 5-Hydroxytryptamine (5-HT)↓, tryptophan hydroxylase-1 (TPH1)↓, 5-hydroxytryptamine 3 receptor (5-HT3r)↓	Decrease of visceral sensitivity	—	Zhao et al. (2020)
	Xiangsha Liujunzi decoction	*Helicobacter* pylori-related gastritis rats	15 mL/kg, 4 weeks	Tumor necrosis factor-alpha (TNF-α)↓, IL-6↓, inducible nitric oxide synthase (iNOS)↓, nitric oxide (NO)↓, mitogen-activated protein kinase (MAPK)↓, nuclear factor kappa-B (NF-κB)↓, TLR2↓, TLR4↓	Reduction of inflammation	—	Lin et al. (2016)
Shenling baizhu powder	Concentrated solution of shenling baizhu powder	Rotavirus enteritis rats, in virtro	8.37 g/kg, 3 days	TLR4↓, Myeloid differentiation primary response gene 88 (MyD88)↓, NF-κB↓, IL-1β↓, IL-6↓, TNF-α↓, IFN-β ↑	Alleviation of inflammation	—	Wang et al. (2021)
​	Shenling baizhu powder	Spleen Qi deficiency rats	0.93, 1.86, and 3.72 g/kg, 7 days	D-xylose content↑, IL-10↑, IL-17↓, Treg cells↑	Regulation of intestinal function and immunity	—	Xiao et al. (2021)
​	Shenling baizhu decoction	Mouse model of diarrhea caused by lard	0.25 g/d, 14 days	MDA↓, SOD↑, LDL↓, TG↓, SS↓, CCK↓, IL-17↓, IL-6↓, SCFAs↑	Improvement of fat metabolism and inflammation	*Lactobacillus reuteri*↑, *Lactobacillus intestinalis*↑	Qiao et al. (2024)
	Shenling baizhu decoction	Pelotinib-induced diarrhea rats	3.6 g/kg, 10 days	Mucosal damage(pathological change)↓, cyclic AMP(cAMP)↑, 25-hydroxycholesterol↑, guanidinosuccinic acid↑, 5-hydroxyindolepyruvate↑	Changes of gut microbiota and its metabolites	*Lachnospiraceae*↑, *Bacilli*↑, *Lactobacillales*↑, *Allobaculum_ stercoricanis*↑, opportunistic bacteria↓	Lai et al. (2022)
	Shenling baizhu decoction	High-fat diet rats	0.75 g/d, 16 weeks	ALT↓, AST↓, T-CHO↓, TG↓, IL-1β↓, IL-18↓, TNF-α↓, LPS↓, TLR4↓, NLRP3↓, MyD88↓, caspase 1↓	Improvement of fat metabolism and inflammation	*Bacteroidetes*/*Firmicutes* ratio↑, *Bifidobacterium*↑, *Akkermansia*↓	Zhang et al. (2018)
	Shenling baizhu decoction	Spontaneously obese type 2 diabetic rats	0.66 g/d, 0.132 g/d, 0.264 g/d, 14 weeks	Glycated hemoglobin(Hb1Ac)↓, TG↓, T-CHO↓, LDL↓, HDL↓, glucose↓	Improvement of fat metabolism, changes of gut microbiota	*Prevotella*↑, *Anaerostipes*↑, *Turicibacter*↑, *Bilophila*↑, *Ochrobactrum*↑, *Acinetobacter*↑, *Lactobacillus*↓, *Roseburia*↓, *Staphylococcusc*↓	Zhang et al. (2021)
Erchen decoction	Erchen decoction	High-fat diet mouse	8.7 g/kg·d, 14 weeks	TG↓, T-CHO↓, PPARγ↑, LPL↑	Improvement of fat metabolism	-	Zhang et al. (2020)
	Concentrated solution of Erchen decoction	Zucker diabetic fatty rats	2.28 g/kg, 4.57 g/kg, 9.14 g/kg, 5 weeks	ITT↓, IRS1↓, AKT↑, PKA↓, HSL↓, TC, HDL↓, LDL-C↓, TG↓	Improvement of fat metabolism, changes of gut microbiota	*Prevotella*↓, *Ruminococcus*↓, *Blautia*↓, *Holdemania*↓	Zhao et al. (2021)
	Erchen decoction	High-fat diet rats	2.28 g/kg, 4.57 g/kg, 9.14 g/kg, 4 weeks	TC↓, TG↓, LDL↓, HDL↓, FFA↓, AST↓, ALT↓, butyric acid↑, HDAC1↓, H3K9ac↑	Improvement of fat metabolism, changes of gut microbiota	*Butyricicoccus*↓, *Bifidobacterium*↓, *Lactobacillus*↓, *Coprobacillus*↑, *Eubacterium*↓	Zhang L. et al. (2023)
	Erchen decoction	High-fat diet mouse	5.7 g/kg, 6 weeks	ALT↓, AST↓, LPS↓, TLR4↓, TNF-α↓, IL-1β↓, NF-κB↓, TG↓, TC↓, FFA↓, tight junction proteins (claudin-3, occludin, and ZO-1)↑, acetic acid↑, propionic acid↑, butyric acid↑	Improvement of fat metabolism and inflammation, changes of gut microbiota	*Firmicutes*↑, *Proteobacteria*↑, *Bacteroidetes*↓, *Cyanobacteria*↓, *Verrucomicrobia*↓	Liu H. et al. (2021)
	Erchen decoction	High-fat diet rats	4.5 g/kg, 9 g/kg, 12 weeks	SOD↑, glutathione peroxidase (GSH-Px)↑, MDA↓, IL-6↓, IL-1β↓, TNF-α↓, ALT↓, AST↓, TG↓, T-CHO↓	Improvement of fat metabolism and inflammation, changes of gut microbiota	*Bacteroidetes*/*Firmicutes* ratio↑, *Lactobacillus*↑, *Dubosiella*↑, *Lachnospiraceae*↑, *Akkermansia*↑, *Intestinimonas*↑, *Desulfovibrio*↓, *C._saccharimonas*↓	Miao et al. (2022)
Daotan decoction	Cangfu daotan decoction	Obese PCOS model rats	1.42 g/kg, 5.68 g/kg, 2 weeks	insulin↑, T-CHO↓, testosterone (T)↓, luteinizing hormone (LH)↓, TG↓, LDL↓, HDL↑, stimulating growth hormone (FSH)↑, estradiol (E2)↑, IL-2↓, IL-6↓, TNF-α↓, OATP3A1↑, OATP2B1↑	Improvement of fat metabolism and inflammation, regulation of hormone level	-	Yi W. et al. (2021)
	Huanglian Wendan decoction	High temperature, high humidity environment, high fat diet rats	7.8 g/kg, 4 weeks	NF-κB↓, NLRP3↓, caspase-1↓, IL-1β↓, IL-18↓, ISI↑, IRI↓, 2-h postprandial glucose (2 hP G)↓	Improvement of inflammation and glycometabolism	-	Dong et al. (2021)
	Huanglian Wendan decoction	Diabetic encephalopathy rats	3 g/kg, 6 g/kg, 30 days	Fasting blood-glucose (FBG)↓, TG↓, T-CHO↓, TNF-α↓, IL-6↓, IL-1β↓, neuron density↑, amyloid deposition↓, AKT↑, IRS-1↑	Improvement of inflammation, fat metabolism and glycometabolism	-	Li et al. (2016)

**TABLE 7:** Summary of clinical studies of TCM formulas.

Formulas	Studies	Intervention	Duration	Participants	Key microbiome	Key factors	Literature
Sijunzi decoction	Observational with intervention (for the treatment group)	Sijunzi decoction	12 weeks	Healthy group, patients with chronic atrophic gastritis (CAG) (CAG group), treatment group	*Erysipelotrichia*↓, *Bacteroides*↓, *Blautia*↓, *Faecalibacterium*↑, *Fusicatenibacter*↑	—	Xing et al. (2024)
Shenling baizhu powder	RCT	Shenling baizhu powder (12 g × 10 bags/box), 18 g/d; Mesalazine enteric-coated tablets (0.25 g)	30 days	Patients (aged 60–78 years) with ulcerative colitis complicated with bloody purulent stool	—	Bloody purulent stool↓, IL-2↓, IFN-γ↓	Li S. et al. (2021)

## 6 The potential mechanism of botanical drugs in treating obesity

### 6.1 Ginseng

Ginseng is a naturally botanical drug. Its ability to invigorate qi and strengthen the spleen is commonly used for both medicinal and dietary applications.

Ginseng extract decreased the abundance of *Firmicutes* (such as *Streptococcus*, *Escherichia-Shigella*, *Veillonella*, *Lactobacillus*, *Bifidobacterium*, *Enterococcus*) in the feces of rats with spleen deficiency syndrome, and also increased the abundance of *Bacteroidetes* (Li et al., 2020). Furthermore, it is also possible to increase the energy metabolism in which brown adipose tissue (BAT) participates by adjusting uncoupling protein 1 (UCP1) and oxidative phosphorylation (OXPHOS). This mechanism is closely related to the increase of *Enterococcus faecalis* (Quan et al., 2020). Red ginseng, a processed product of ginseng, its extract can regulate the GBA (such as vasoactive intestinal peptide (VIP), acetylcholinesterase (AChE), and 5-HT) and energy metabolism (such as adrenocorticotropic hormone (ACTH), cortisol (CORT), Na+-K+-ATPase, and citrate synthase (CS)), increase *Bacteroidetes*/*Firmicutes* ratio and *Lactobacillus*, decrease *Akkermansia*, and also alter SCFAs levels. Moreover, the brain-gut axis-related indicators are closely related to changes in the gut microbiota (Zhang X. et al., 2023). Not only that, Protopanaxadiol ginsenosides can increase beneficial bacteria (*Prevotella_9*, *Faecalibacterium*, *Dialister*), and reduce harmful bacteria (*Escherichia-Shigella*, *Dorea*, *Lachnoclostridium*) (Zhang et al., 2021). Ginsenoside Rk3, for one, can increase the *Bacteroidetes*/*Firmicutes* ratio, *Actinomycetes*, *Bifidobacteria*, and *Lactobacilli*, by inhibiting the TLR4/MyD88/NF-κB pathway to reduce inflammatory factor expression and alleviate intestinal mucosal damage (Chen et al., 2021). Bai et al. also confirmed in dysbiosis mice that its function in modulating the gut microbiota and improving inflammation and mucosal damage can also increase the content of SCFAs (Bai et al., 2021). It can also modulate lipid metabolism and enhance anti-inflammatory responses (Wang et al., 2024). Another ginsenoside, Ginsenoside F2, can downregulate pro-inflammatory cytokine genes (*Il1b*, *Tnf*, and *Il6*) in high-fat mice and modulate liver X receptor (LXR)-related gene expression (*Srebf1* and *Fasn)*, improving lipid metabolism and inflammatory responses (Kim et al., 2024). Another study found that it can increase AMPK, inhibit ACC, and improve fat metabolism (Zhou et al., 2021).

Kaempferol, a flavonoid in ginseng, It can downregulate the expression of inflammatory factors in Leptin receptor-deficient obese mice through the NLRP3 pathway (Zhai et al., 2024). It can also enhance intestinal barrier function and reduce inflammation in high-fat diet-fed mice by suppressing the TLR4/NF-κB pathway, and by increasing the *Bacteroidetes*/*Firmicutes* ratio while reducing the abundance of genera such as *Alistipes*, *Lachnospiraceae_NK4A136_group*, and *Romboutsia*. Furthermore, it was discovered that changes in the gut microbiota were closely associated with the levels of inflammation (Bian et al., 2022). Moreover, it can also adjust lipid metabolism, reduce *Firmicutes* in the gut microbiota, and increase *Bacteroidetes*, *Proteobacteria*, and *Akkermansia* (Wang et al., 2020).

Therefore, Ginseng and its metabolites not only participate in regulating the inflammatory response and glycolipid metabolism caused by obesity, but can also improve gut microbiota dysbiosis.

### 6.2 Atractylodes macrocephala Koidz

Atractylodes macrocephala Koidz, a widely used botanical drug in TCM, can strengthen the spleen, enhance qi, alleviate dampness, and promote urination. Polysaccharides from Atractylodes macrocephala Koidz can reduce ferroptosis-related gene expression and improve the inflammatory response (Li W. et al., 2022). Moreover, it can increase the beneficial bacterial *Bacteroides* and *Lactobacillus* in the intestine, and improve mucosal damage in mice with colitis (Zhang et al., 2025). Kai et al. found that it can not only alleviate colon mucosal damage and inflammatory responses, but also effectively reduce harmful bacteria such as *Clostridium sensu stricto 1* and *Escherichia Shigella* (Kai et al., 2022). Therefore, the main function of polysaccharides is to regulate gut microbiota imbalance, ensure the stability of the intestinal mucosal barrier, and improve the inflammatory response.

Atractylenolide I and III, key bioactive metabolites in Atractylodes macrocephala Koidz. Atractylenolide I can enhance *Lactobacillus* and *Bacteroides*, reduces the levels of *Escherichia* and *Candidatus*, and by suppressing the TLR4/MyD88/NF-κB pathway to reduce IL-1β and LPS levels (Liu P. et al., 2021). Atractylenolide III can downregulate nuclear respiratory factor 2 (Nrf2), NADPH oxidase 1 (NOX1), formyl peptide receptor (FPR) 1 and dual oxidase 2 (DUOX2), downregulate the expression of IL-1β and TNF-α, and also suppresses *Actinobacteria* growth, elevates *Bacteroidetes* levels (Ren et al., 2021).

In summary, Atractylodes macrocephala Koidz can modulate inflammatory responses and oxidative stress, reduce harmful intestinal bacteria, and increase beneficial bacteria.

### 6.3 Chinese yam

Jeon et al. found that Chinese yam extract can reduce neutral lipids and *Bacteroides fragilis* (Jeon et al., 2006). Cui et al. found that Chinese yam extract can increase *Clostridium*, *Lactobacillus*, and *Akkermansia*, and increase SCFAs levels. At the same time, it was found that polysaccharides, diosgenin, and taxifolin are the metabolites of Chinese yam (Cui et al., 2023). Moreover, it can also reduce the IL-1β and IL-6 levels in diarrhea mice, while simultaneously increasing *Bacteroides thetaiotaomicron* and *Paramuribaculum intestinale* (Pan et al., 2022).

Diosgenin, an active metabolite of Chinese yam, can modulate immunoregulation involving CD4 and CD8 cells, upregulate IFN-γ levels, and increase the levels of *Lactobacillus*, *Sutterella*, and *Bacteroides* (Dong et al., 2018). He et al. found that it can also regulate the SCFAs levels in colitis mice, increase the abundance of *Prevotella*, *Odoribacter*, *Mucispirillum* and *Veillonella* (He et al., 2022). These changes may be related to its improvement of mouse intestinal mucosal injury. Furthermore, Diosgenin can also regulate BA metabolism by downregulating CYP7A1 through upregulating FXR and fibroblast growth factor (FGF) 15, and this mechanism is participated in by *Clostridia* (Yan et al., 2023). Taxifolin, another metabolite, participates in regulating the fat metabolism of mice on a high-fat diet, boosts SOD activity, while increasing the *Bacteroidetes*/*Firmicutes* ratio and reducing the levels of harmful bacteria *Mucispirillum* and *Desulfovibrio* spp. (Su et al., 2022). Stigmasterol, also found in Chinese yam, can improve immune and inflammatory responses, while simultaneously increasing SCFA content and regulating the gut microbiota. Furthermore, FMT confirmed that the microbiota participates in the regulation of immune and inflammatory responses (Wen et al., 2021). It is also possible to improve lipid metabolism by reducing the expression levels of NLRP3 and Caspase-1 and increasing CYP7B1, while simultaneously downregulating IL-18 and IL-1β, and reducing the harmful bacteria *Lachnospiraceae_NK4A136_group*, *Desulfovibrio*, and *Lactobacillus* (Xin et al., 2023).

Thus, Chinese yam and its active compounds can participate in regulating inflammation and immune responses, modulating BA metabolism, and these improvements are related to gut microbiota adjustment.

### 6.4 Poria cocos

Poria cocos, initially documented in *Shennong’s Herbal Classic*, effectively promotes urination, reduces edema, fortifies the spleen, and calms the mind. Sun et al. found that its insoluble polysaccharide metabolites can significantly improve glucose and lipid metabolism, reduce inflammation, while simultaneously increasing SCFA-producing related microbial communities (such as *Clostridum IV*, *Ruminococcus*, and *Bacteroides*) and reducing harmful bacteria such as *Megamonas* and *Proteus* (Sun et al., 2019). And Poria cocos oligosaccharides can modulate BA metabolism and inflammation levels in obese mice, with this alteration being associated with the participation of the gut microbiota (Zhu et al., 2022).

In summary, poria cocos can modulate BA metabolism, lipid metabolism, and inflammation, which are processes involved in changes in the gut microbiota.

### 6.5 Glycyrrhiza

Glycyrrhiza is a Chinese medicine, and TCM believes that it can invigorate qi, which is often used in combination with other Chinese medicines.

Research indicates that Glycyrrhiza extract modulates lipid and glucose metabolism in diabetic mice. It reduces inflammation mediated by IL-6, IL-12, and TNF-α through the TLR4/NF-κB pathway and ameliorates intestinal mucosal injury. This is associated with an increase in *Alloprevotella* and *Bacteroides*, and a decrease in *Lachnospiraceae_NK4A136_group* (Zhang Y. et al., 2022). Liu et al. also discovered that the extract of Glycyrrhiza can participate in regulating fat metabolism and alleviating inflammation, and it is closely related to the decrease of *Clostridium sensu stricto 1* and *Lactobacillus* (Liu F. et al., 2022).

Glabridin and isoliquiritigenin are the main metabolites of Glycyrrhiza and are involved in the mechanisms of obesity improvement. Glabridin can alleviate inflammatory responses by down-regulating NF-κB expression and increasing STAT6 activity; in this process, the *Bacteroidetes*/*Firmicutes* ratio and *Desulfovibrio* decrease, while *Lactobacillus* increases (Huang et al., 2019). Isoliquiritigenin can reduce the expression of monocyte chemoattraction protein-1 (MCP-1), F4/80, and CD11c in mice fed a high-fat diet, ameliorate white adipose tissue (WAT) generation and inflammatory responses, and decrease *Bacteroides spp*, *Lactobacillus johnsonii*, *Lactococcus lactis*, and *Firmicutes* (Ishibashi et al., 2022).

In summary, Glycyrrhiza and its metabolites modulate fat metabolism, reduce inflammation, and improve gut microbiota imbalance.

### 6.6 Rhizoma Alismatis

Rhizoma Alismatis (*Alisma orientale *(Sam.) Juzep. raw dried rhizome) is commonly used to treat diseases, including edema and hyperlipidemia. Xu et al. found that Rhizoma Alismatis extract can downregulate the total cholesterol (T-CHO) and low-density lipoprotein levels in high-fat diet rats, and reduce *Lactobacillus* (Xu et al., 2020). Alisol A 24-acetate (AA-24-a), a key triterpenoid in Rhizoma Alismatis, can change fat metabolism-related genes (downregulate HSL, PPARγ, and perilipin A, upregulate adipose triglyceride lipase (ATGL)) (Lou et al., 2021). However, the relationship between this mechanism and gut microbiota lacks relevant research.

### 6.7 Crataegus pinnatifida Bung

Crataegus pinnatifida Bunge (*Crataegus pinnatifida* Bge. var. *major* N. E. Br. raw dried ripened fruit) was recorded as early as *the Annotation of Materia Medica* and is mainly used to treat anorexia, hyperlipidemia, hypertension, and other diseases in the clinic.

Procyanidins, quercetin, naringenin are metabolites extracted from Crataegus pinnatifida Bunge and are involved in regulating fat and glucose metabolism. Procyanidins can reduce inflammation and regulate fat metabolism by reducing the TLR4/NF-κB pathway and increasing the AMPK pathway, and they are associated with a decrease in *Akkermansia* and *Bacteroides* and an increase in *Bifidobacterium*, *Blautia*, *Lachnospiraceae*, and *Subdoligranulum* (Han et al., 2022). Moreover, it can also improve the inflammatory response in gestational diabetes mellitus mice through the NF-κB/NLRP3 pathway (Liu Y. et al., 2022). Quercetin combined with resveratrol can downregulate IL-6 and TNF-α in rats fed a high-fat diet, increase the *Bacteroidetes*/*Firmicutes* ratio, *Ruminococcaceae_UCG-014* and *Ruminococcaceae_UCG-005*, and decrease *Lachnoclostridium* (Zhao et al., 2017). Naringenin can regulate genes involved in energy (TFAM, NRF1/2, UCP1, PGC1α and TFAM) and fat metabolism (CD137, HOXC8 and TBX1), increase *Akkermansia*, adjust SCFAs levels (particularly decrease acetic acid), improve obesity (Zhang S. et al., 2022).

Therefore, Crataegus pinnatifida Bunge and its metabolites may be able to ameliorate obesity by modulating fat and glucose metabolism and regulate gut microbiota.

### 6.8 Pericarpium citri reticulatae

Pericarpium citri reticulata is a TCM. It has significant medicinal properties and a long history of use in both food and medicine. Studies have shown that it regulates lipid metabolism. Research revealed that its extract can participate in the regulation of fat metabolism, and is closely related to *Ruminococcaceae* and *Rikenellaceae*, while also being able to reduce *Firmicutes*, increase *Bacteroides*, and also increase the abundance of *Anaerotruncus*, *Odoribacter*, *Rikenellaceae_RC9_gut_group*, *Alistipes*, and *Ruminiclostridium_9* (Li A. et al., 2021).

Polymethoxyflavones (PMFs) and hydroxyl PMFs (HPMFs) are the active metabolites of pericarpium citri reticulatae. Subsequent research has indicated that PMFs and HPMFs can diminish adipocytes in perigonadal fat by downregulating adiponectin 1 protein, and sterol regulatory element binding protein 1 (SREBP-1) expression in obese mice. *Prevotella* and *rc4-4* also decreased (Tung et al., 2018). Moreover, Hesperidin and tangeretin as flavonoids in pericarpium citri reticulatae, can participate in the regulation of gut microbiota and lipid metabolism. Hesperidin can regulate lipid metabolism (upregulating PPARα, CPT1α, ACOX1, and ACADM, downregulating HADH, SREBP-1C, and SCD1) and the gut microbiota (mainly increasing the *Bacteroidetes*/*Firmicutes* ratio), participating in the regulation of lipid metabolism (Li X. et al., 2022). Tangeretin can activate Nrf2, thereby downregulating NF-κB and IκB-α to improve the inflammatory response in mice on a high-fat diet, regulate fat metabolism, and also alter the gut microbiota (increasing the *Firmicutes*/*Bacteroidetes* ratio and the abundance of *Bacteroidetes* and *Lactobacillus*) (Chen et al., 2022).

In summary, the research found that botanical drugs and their active metabolites can participate in regulating the gut microbiota and SCFAs levels, improving bile acid metabolism, modulating related hormones of the BGA, and regulating inflammatory factors.

The specific mechanisms of botanical drugs and active metabolites for strengthening the spleen and dispelling dampness are shown in Tables 8, 9.

**TABLE 8:** Summary of pre-clinical studies of botanical drugs.

Botanical drugs	Intervention	Model	Dosages	Target/Factors	Mechanism	Gut microbiota	Literature
Ginseng	Ginseng extracts	*In vitro*	0.4 mg/mL, 48 h	—	Changes of gut microbiota	*Streptococcus*↓, *Escherichia-Shigella*↓, *Veillonella*↓, *Lactobacillus*↓, *Bifidobacterium*↓, *Enterococcus*↓, *Actinobacteria*↓, *Bacteroidetes*↑	Li et al. (2020)
	Ginseng extracts	High-fat diet mouse, FMT, antibiotic depletion	10 mg/kg, 2 weeks	Brown adipose tissue (BAT)↑, UCP1↑, OXPHOS↑	Improvement of fat metabolism, changes of gut microbiota	*Enterococcus*(Genus)↑, *Enterococcus faecalis*(Species)↑	Quan et al. (2020)
	Red ginseng extracts	Spleen qi deficiency rats	3.24 g/kg, 6.48 g/kg, 10 days	D-xylose↑, VIP↓, SP↑, AChE↑, ACTH↑, CORT↑, T3↑, T4↑, E2↑, 5-HT↑, CS↑, NCR↑, IDH1↑, COX↑, Na + -K + -ATPase↑, cAMP↑, cGMP↓, acetic acid↓, propionic acid↓, isobutyric acid↑, butyric acid↑, isovaleric acid↑	Changes of gut microbiota, regulation of GBA and energy metabolism	*Bacteroidetes*/*Firmicutes*↑, *Lactobacillus*↑, *Akkermansia*↓	Zhang X. et al. (2023)
	Protopanaxadiol ginsenosides	*In vitro*	15 mg/mL, 24 h	—	Changes of gut microbiota	*Prevotella_9*↑, *Faecalibacterium*↑, *Dialister*↑, *Escherichia-Shigella*↓, *Dorea*↓, *Lachnoclostridium*↓	Zhang et al. (2021)
Atractylodes macrocephala Koidz	Atractylodes macrocephala Koidz	Ferroptosis goslings	400 mg/kg, 28 days	Ferroptosis genes (GPX4, FTH1, FPN1, HSPB1, COX-2, NOX1, TFR1, ACSL4)↓, IFN-γ↓, IL-1β↓, IL-4↓, IL-6↓, IL-10↓, IL-17↓, IL-18↓, TNF-α↓	Improvement of ferroptosis and inflammation	*—*	Li W. et al. (2022)
	Atractylodes macrocephala Koidz	DSS-induced colitis mouse	100 mg/kg, 14 days	ZO-1↑, claudin↑	Changes of gut microbiota	*Bacteroides*↑, *Lactobacillus*↑	Zhang et al. (2025)
	Atractylodes macrocephala Koidz	DSS-induced colitis mouse	100 mg/kg, 200 mg/kg, 400 mg/kg, 15 days	IL-1β↓, IL-6↓, TNF-α↓, ZO-1↑, claudin-1↑, Occludin↑, MUC-2↑	Improvement of inflammation, changes of gut microbiota	*Clostridium sensu stricto 1*↓, *Escherichia Shigella*↓	Kai et al. (2022)
Chinese yam	Chinese yam ethanol extract	SD rats	Add 2% or 10% to food, 6 weeks	Neutral lipids↓	Changes of gut microbiota	*Bacteroides fragilis*↓	Jeon et al. (2006)
	Chinese yam extract	*In vitro*	24 h	Acetic acid↑, butyric acid↑	Changes of gut microbiota	*Clostridium*↑, *Lactobacillus*↑, *Akkermansia*↑	Cui et al. (2023)
	Chinese yam	Antibiotic-associated diarrhea mouse	30 mg/kg, 14 days	IL-1β↓, IL-6↓	Improvement of inflammation, changes of gut microbiota	*Bacteroides thetaiotaomicron*↑, *Paramuribaculum intestinale*↑	Pan et al. (2022)
Poria cocos	Water insoluble polysaccharide	Ob/ob mice	1 g/kg, 0.5 g/kg, 4 weeks	T-CHO↓, TG↓, LDL↓, SOD↑, TNF-α↓, LPS↓, ISI↑, glucose↓, insulin↓, AST↓, ALT↓	Improvement of fat metabolism and inflammation, changes of gut microbiota	*Lachnospiracea*↑, *Alloprevotella*↑, *Parabacteroides*↑, *Clostridum IV*↑, *Ruminococcus*↑, *Bacteroides*↑, *Megamonas*↓, *Proteus*↓	Sun et al. (2019)
	Poria cocos oligosaccharides powder	High-fat diet mouse, FMT, antibiotic depletion	200 mg/kg, 8 weeks	Glucose tolerance test↓, insulin tolerance test↓, insulin↓, TNF-α↓, IL-1β↓, IL-6↓, COX-5b↓, MCP-1↓, GPR43↓, NLRP3↓, FXR↓, FGF15↓,CA↑, UDCA↑, valeric acid↓, 5-HT↓	Improvement of fat metabolism and inflammation, changes of gut microbiota	*Bacteroidetes*/*Firmicutes*↑, *Ruminococcaceae*↓, *Anaeroplasmataceae*↓, *Lactobacillaceae*↑, *Rikenellaceae* ↑	Zhu et al. (2022)
Glycyrrhiza	Glycyrrhiza extract	Type 2 diabetes mice	20 mg/kg, 40 mg/kg, 80 mg/kg, 4 weeks	Insulin↓, LPS↓, insulin resistance↓, HDL↑, TG↓, LDL↓, T-CHO↓, ALT↓, AST↓, occludin↑, ZO-1↑, IL-6↓, IL-12↓, TNF-α↓, NF-κB↓, TLR4↓, IKKα↓	Improvement of fat metabolism and inflammation, changes of gut microbiota	*Bacteroidetes*↑, *Firmicutes*↓, *Alloprevotella*↑, *Bacteroides*↑, *Lachnospiraceae_NK4A136_group*↓, *Akkermansia*↑	Zhang Y. et al. (2022)
	Glycyrrhiza extract	High-fat diet mouse, antibiotic depletion, *in vitro*	0.2 mL, 4 weeks	AST↓, alkaline phosphatase (ALP)↓, cholinesterase (CHE)↓, TG↓, T-CHO↓, LDL↓, TNF-α↓, IL-6↓, IL-10↑, fatty acid synthase (FASN)↑, acylglycerol lipase (MGL)↑, SCFAs↓	Improvement of fat metabolism, changes of gut microbiota	*Clostridium sensu stricto 1*↓, *Lactobacillus*↓	Liu F. et al. (2022
Rhizoma Alismatis	Rhizoma Alismatis water extract	High-fat and high-sugar diet rats	2.1 g/kg, 14 days	T-CHO↓, LDL↓	Improvement of fat metabolism and inflammation, changes of gut microbiota	*Lactobacillus*↓	Xu et al. (2020)
Pericarpium citri reticulatae	Pericarpium citri reticulatae extract	High-fat diet mouse	5 g/kg, 10 g/kg, 12 weeks	T-CHO↓, TG↓, HDL↑, LDL↓	Improvement of fat metabolism and inflammation, changes of gut microbiota	*Firmicutes*↓, *Bacteroides*↑, *Anaerotruncus*↑, *Odoribacter*↑, *Rikenellaceae_RC9_gut_group*↑, *Alistipes*↑, *Ruminiclostridium_9*↑	Li A. et al. (2021)

**TABLE 9:** Summary of pre-clinical studies of metabolites.

Botanical drug	Metabolites	Structural formulas	Intervention	Model	Dosages	Target/factors	Mechanism	Gut microbiota	Literature
Ginseng	Ginsenoside Rk3	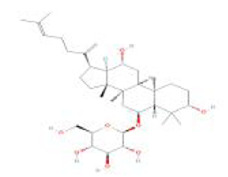	Ginsenoside Rk3	High-fat diet mouse	30 mg/kg, 60 mg/kg, 7 weeks	IL-6↓, TNF-α↓, IL-1β↓, LPS↓, glucose tolerance↓, ZO-1↑, occludin↑, claudin↑, TLR4↓, MYD88↓, NF-κB↓, IκB-α↑, total SCFAs↑	Improvement of inflammation, changes of gut microbiota	*Bacteroidetes*/*Firmicutes*↑, *Actinomycetes*↑, *Bifidobacteria*↑, *Lactobacilli*↑	Chen et al. (2021)
				Antibiotic-induced gut microbiota dysbiosis mouse	20 mg/kg, 60 mg/kg, 2 weeks	IL-6↓, TNF-α↓, IL-1β↓, IL-17↓, IL-10↑, IFN-γ↑, ZO-1↑, occludin↑, claudin↑,acetate↑, propionate↑, butyrate↑	Improvement of inflammation, changes of gut microbiota	*Bacteroidetes*/*Firmicutes*↑	Bai et al. (2021)
				High-fat diet mouse	30 mg/kg, 60 mg/kg, 8 weeks	MDA↓, SOD↑, LDL↓, HDL↑, TG↓, T-CHO↓, PGE2↓, PGD2↓, TXB2↓, HETE↓, HODE↓	Improvement of inflammation, changes of gut microbiota	*—*	Wang et al. (2024)
	Ginsenoside F2	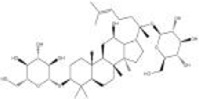	Ginsenoside F2 purified from yeast	Wild type and LXRα deficient mouse with a high-fat diet	50 mg/kg, 12 weeks	ALT↓, AST↓, Srebf1↓, Fasn↓, Il1b↓, Tnf↓, Il6↓	Improvement of fat metabolism and inflammation	*—*	Kim et al. (2024)
			Ginsenoside F2	*In vitro*	-	AMPK↑, ACC↓, T-CHO↓, TG↓, AST↓	Improvement of fat metabolism	*—*	Zhou et al. (2021)
	Kaempferol	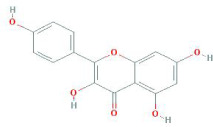	Kaempferol	Leptin receptor-deficient obese mice	50 mg/kg, 6 weeks	Glucose tolerance test↓, insulin tolerance test↓, F4/80↓, TNF-α↓, IL-18↓, IL-10↓, NLRP3↓, caspase-1↓	Improvement of inflammation	*—*	Zhai et al. (2024)
			Kaempferol	High-fat diet mouse	High fat diet with 0.1% kaempferol, 16 weeks	T-CHO↓, TG↓, TNF-α↓, IL-1β↓, IL-6↓,myeloperoxidase (MPO),TLR4↓, MyD88↓, NF-κB↓,F4/80↓	Improvement of inflammation and fat metabolism, changes of gut microbiota	*Bacteroidetes*/*Firmicutes*↑, *Alistipes*↓, *Lachnospiraceae_NK4A136_group*↓, *Romboutsia*↓, *Faecalibaculum*↓, *Kaempferol*↓	Bian et al. (2022)
			Kaempferol	High-fat diet mouse	200 mg/kg, 8 weeks	TG↓, glucose↓, HDL↑, LDL↓, T-CHO↓	Improvement of fat metabolism, changes of gut microbiota	*Firmicutes*↓, *Bacteroidetes*↑, *Proteobacteria*↑, *Akkermansia*↑	Wang et al. (2020)
Atractylodes macrocephala Koidz	Atractylenolide I	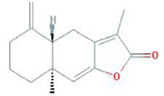	Atractylenolide I	Antibiotic-induced gut microbiota dysbiosis mouse, *in vitro*	-	LPS↓, IL-1β↓,TLR4↓, MyD88↓, NF-κB↓	Improvement of inflammation, changes of gut microbiota	*Lactobacillus*↑, *Bacteroides*↑,*Escherichia*↓, *Candidatus*↓	Liu P. et al. (2021)
	Atractylenolide III	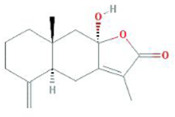	Atractylenolide III	TNBS-induced colitis moiuse	5 mg/kg, 10 mg/kg, 20 mg/kg, 14 days	FPR1↓, DUOX2↓, NOX1↓, Nrf2↓, MDA↓, SOD↑, GSH-Px↑, TNF-α↓, IL-1β↓	Improvement of inflammation, changes of gut microbiota	*Actinobacteria*↑, *Bacteroidetes*↑	Ren et al. (2021)
Chinese yam	Diosgenin	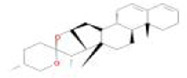	Diosgenin	Melanoma-bearing mouse, *in vitro*	20 mg/kg, 14 days	CD4 cells↑, CD8 cells↑, IFN-γ↑	Immunoregulation, changes of gut microbiota	*Lactobacillus*↑, *Sutterella*↑, *Bacteroides*↑	Dong et al. (2018)
			Diosgenin	DSS induced colitis mouse	15 mg/kg, 7 days/14 days	Total SCFAs↑,acetic acid↑, propionic acid↑, isobutyric acid↑	Changes of gut microbiota	*Prevotella*↑, *Odoribacter*↑, *Mucispirillum*↑, *Veillonella*↑	He et al. (2022)
			Diosgenin	Methionine and choline-deficient (MCD) feeding mouse, FMT	30 mg/10 mL/kg, 1 week	TG↓, T-CHO↓, ALT↓, AST↓, FXR↑, CYP7A1↓, fibroblast growth factor(FGF) 15↑	Improvement of BAs and fat metabolism, changes of gut microbiota	*Clostridia*↑	Yan et al. (2023)
	Taxifolin	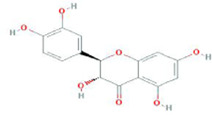	Taxifolin	High-fat diet mouse	0.5 mg/mL, 1 mg/mL, 15 weeks	SOD↑, TG↓, T-CHO↓, HDL↑, LDL↓,MDA↓, SOD↑,GSH-Px↓	Improvement of fat metabolism, changes of gut microbiota	*Bacteroidetes*/*Firmicutes*↑, *Mucispirillum*↓, *Desulfovibrio spp*↓	Su et al. (2022)
	Stigmasterol	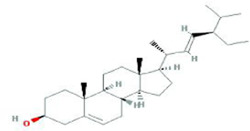	Stigmasterol	DSS induced colitis mouse, antibiotic depletion, FMT	400 mg/kg, 10 days	IL-6↓, IL-1β↓, TNF-α↓, IL-10↑, IL-17A↓,acetate↑, propionate↑, butyrate↑, isobutyrate↑, valerate↑	Improvement of inflammation, changes of gut microbiota	*Helicobacter*↑, *Odoribacter*↑, *Prevotella*↑, *Oscillospira*↑, *Paraprevotella*↑, *Turicibacter*↑, *Ruminococcus*↑, *Butyricicoccus*↑, *Ruminococcaceae*↑, *and Paraprevotellaceae*↑	Wen et al. (2021)
			Stigmasterol	High-fat and high-cholesterol diet mouse	200 mg/kg, 10 weeks	NLRP3↓, IL-18↓, T-CHO↓, TG↓, HDL↑, Caspase-1↓, IL-1β↓, CYP7B1↑	Improvement of inflammation, changes of gut microbiota	*Lachnospiraceae_NK4A136_group*↓, *Desulfovibrio*↓, *Lactobacillus*↓	Xin et al. (2023)
Glycyrrhiza	Glabridin	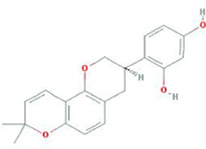	Glabridin	DOX-induced cardiotoxicity mouse	15 mg/kg, 30 mg/kg, 12 days	Caspase-3↓, caspase-9↓, HAX-1↑, Bcl-2↑, LPS↓, IL-1β↓, TNF-α↓, IL-10↑, TGF-β↑, NF-κB↓, signal transducer and activator of transcription 6 (STAT6)↑	Improvement of inflammation, changes of gut microbiota	*Bacteroidetes*/*Firmicutes*↓, *Desulfovibrio*↓, *Lactobacillus*↑	Huang K. et al. (2019)
	Isoliquiritigenin	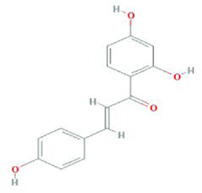	Isoliquiritigenin	High-fat diet mouse, FMT	2 weeks	glucose↓, TNF-α↓, monocyte chemoattractant protein-1 (MCP-1)↓, F4/80↓, CD11c↓	Improvement of inflammation and fat metabolism	*Bacteroides spp*↓, *Lactobacillus johnsonii*↓, *Lactococcus lactis*↓, *Firmicutes*↓	Ishibashi et al. (2022)
Rhizoma Alismatis	Alisol A 24-acetate	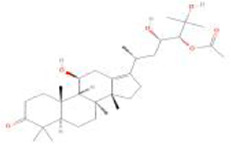	Alisol A 24-acetate	*In vitro*	10–50 μM, 2 days	HSL↓, adipose triglyceride lipase (ATGL)↑, PPARγ↓, perilipin A↓	Improvement of fat metabolism	*—*	Lou et al. (2021)
Crataegus pinnatifida Bung	Procyanidins	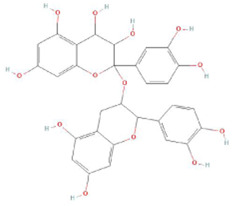	Procyanidins	Lipid metabolism disorder mouse	50 mg/kg, 100 mg/kg, 200 mg/kg, 8 weeks	T-CHO↓, TG↓, LD↓L, HDL↓, SOD↑, MDA↓, glutathione (GSH)↑, ALT↓, AST↓, insulin↓, LPS↓, adiponectin (ADPN), leptin, TLR4↓, NF-κB↓, MYD88↓, IKKβ↓, TNF-α↓, IL-1β↓, AMPK↑, Acc↑	Improvement of inflammation	*Akkermansia*↓, *Bacteroides*↓, *Bifidobacterium*↑, *Blautia*↑, *Lachnospiraceae*↑, *Subdoligranulum*↑	Han et al. (2022)
			Procyanidins	Gestational diabetes mellitus mouse, feeding a high-fat-high-sucrose diet	27.8 mg/kg, 4 weeks	IL-6↓, TNF-α↓, IL-17↓, NF-κB↓, NLRP3↓	Improvement of inflammation	*—*	Liu Y. et al. (2022)
	Quercetin	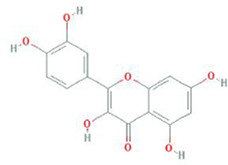	Combination of quercetin and resveratrol	High-fat diet mouse	30 mg/kg, 10 weeks	IL-6↓, TNF-α↓, MCP-1↓	Improvement of inflammation, changes of gut microbiota	*Bacteroidetes*/*Firmicutes*↑, *Lachnoclostridium*↓, *Ruminococcaceae_UCG-014*↑, *Ruminococcaceae_UCG-005*↑	Zhao et al. (2017)
	Naringenin	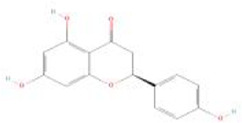	Naringenin	High-fat diet rats	100 mg/kg, 12 weeks	WAT↓, O2 consumption↑, CO2 production↑, energy expenditure↑, beige adipose browning related-markers (CD137, HOXC8 and TBX1)↑, mitochondrion related markers (TFAM, NRF1/2, UCP1, PGC1α and TFAM)↑, acetic acid↑	Changes in energy and fat metabolism, changes of gut microbiota	*Akkermansia*↑	Zhang S. et al. (2022)
Pericarpium citri reticulatae	Polymethoxyflavones	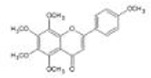	Polymethoxyflavones and hydroxyl polymethoxyflavones	High-fat diet mouse, *in vitro*	25 μg/mL, 50 μg/mL, 2 days	SREBP-1↓, perilipin 1↓, lipid↓	Changes in fat metabolism, changes of gut microbiota	*Prevotella*↓, *rc4-4*↓	Tung et al. (2018)
	Hydroxyl polymethoxyflavones	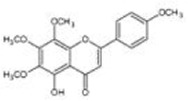							
	Hesperidin	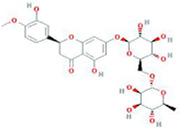	Hesperidin	High-fat diet mouse	0.2% wt/wt in diet, 16 weeks	TG↓, T-CHO↓, LDL↓, AST↓, ALT↓, fatty acid oxidation gene (PPARα↑, CPT1α↑, ACOX1↑, ACADM↑, HADH↓, SREBP-1C↓, SCD1↓)	Changes in fat metabolism and inflammation, changes of gut microbiota	*Bacteroidetes*/*Firmicutes*↑,*Bacteroides*↑, *Prevotellaceae*↑, *Bacteroides_sartorii*↑	Li X. et al. (2022)
	Tangeretin	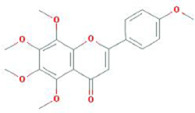	Tangeretin	High-fat diet mouse	100 mg/kg, 12 weeks	ALT↓, AST↓, T-CHO↓, TG↓, TNF-α↓, IL-6↓, LPS↓, Nrf2↑, NF-κB↓, IκB-α↓	Changes in fat metabolism, changes of gut microbiota	*Bacteroidetes*/*Firmicutes*↑, *Bacteroides*↑, *Lactobacillus*↑	Chen et al. (2022)

## 7 Conclusion and prospect

Contemporary approaches to tackling obesity typically involve holistic lifestyle management, including dietary adjustments, regular physical activity, and behavioral therapies. While this strategy is typically regarded as both safety and scalability, its efficacy differs among individuals. This process also necessitates a focus on patient adherence, sustaining its implementation poses a significant challenge. Pharmaceutical interventions and surgical procedures offer alternative avenues, but often result in undesirable side effects.

TCM is an ancient oriental medicine that has long been proposed as a method to treat obesity. TCM combines traditional sports such as *Tai Chi* with acupuncture and botanical drugs to treat obesity. It also formulates corresponding treatment plans based on different personal characteristics. Therefore, TCM is increasingly accepted as a viable approach for managing obesity. TCM emphasizes that spleen deficiency and excess dampness are the root causes of obesity, highlighting their impact on energy metabolism. TCM treatment of obesity focuses on invigorating the spleen and eliminating dampness to adjust energy metabolism. Therefore, we focused on formulas and botanical drugs that strengthen the spleen and dispel dampness.

**FIGURE 3:**
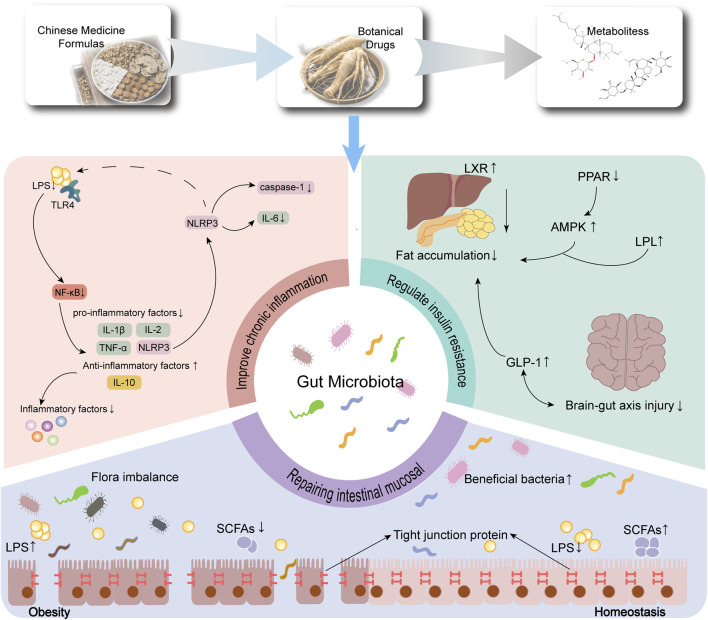
Common Potential Mechanism of TCM formulas and botanical drugs that strengthen the spleen and dispel dampness in improving obesity. Notes: The common mechanism involves maintaining gut barrier function, reducing chronic inflammation, and enhancing insulin-regulated energy metabolism. Abbreviations: NF-κB in the figure refers to the NF-κB pathway; NLRP3 in the figure refers to the NLRP3/caspase-1 pathway; AMPK: in the figure refers to the AMPK pathway. TLR4: toll-like receptor 4; LPS: lipopolysaccharide; SCFAs: short-chain fatty acids; GLP-1: glucagon-like peptide 1; TNF-α: tumor necrosis factor-α; IL-6: interleukin-6; IL-2: interleukin-2; IL-10: interleukin-10; IL-1β: interleukin-1β; LXR: liver X receptor; PPAR γ: peroxisome proliferator-activated receptor γ; LPL: lipoprotein lipase.

Research indicates that TCM formulas and botanical drugs that strengthen the spleen and dispel dampness, with the participation of the gut microbiota, can regulate sugar and lipid metabolism, alleviate inflammation, maintain intestinal barrier function, and improve obesity and its associated metabolic disorders (Figure 3 illustrates the common potential mechanisms of TCM formulas and botanical drugs for obesity treatment).

These formulas, which strengthening the spleen and dispelling dampness, are recorded in ancient Chinese medical literature to treat obesity and are often used clinically. Although plant-derived natural therapies have long been considered safe, economical, and effective, the field currently faces the challenge of insufficient clinical validation data. While numerous animal experiments and *in vitro* studies have provided preliminary evidence for the feasibility of these metabolites, their actual efficacy and bioavailability in the human body still require more rigorous, large-scale clinical trials to be confirmed.

It is particularly noteworthy that while existing research has confirmed the efficacy of TCM formulas, botanical drugs, and their active metabolites in multiple disease models, it has also preliminarily indicated the regulatory role of the gut microbiota in this process. It has been confirmed that NF-κB and AMPK are the primary pathways through which the gut microbiota regulates inflammatory responses, and they can mediate macrophage-related inflammatory factor expression, such as IL-6 and TNF-α. Changes in the levels of SCFAs (such as acetic acid, propionic acid, butyric acid, etc.) are also a significant characteristic of obesity improvement. These studies have also clarified that formulas, botanical drugs, and active metabolites can help improve intestinal mucosal damage and alleviate LPS leakage. They can also participate in regulating BA metabolism through BA-related signaling pathways (such as FXR, FGF15, CYP7A1). They can also regulate obesity-related hormone levels through GBA, regulate insulin resistance, and improve glucose and lipid metabolism.

However, current research is insufficient to clarify the exact causal relationship between these mechanisms and the gut microbiota. In particular, future research will focus on, and it is a key link to understanding their mechanisms of action, whether the overall dysbiosis of the gut microbiota plays a dominant role or whether specific dominant bacterial communities or their metabolites play a key role in the process of formulas, botanical drugs, or active metabolites exerting their effects. This requires further FMT experimental verification.

Formulas, botanical drugs, and their active metabolites have demonstrated significant therapeutic potential in the treatment of obesity. Furthermore, translational pharmacology plays an indispensable role in modernizing and developing TCM. While reviewing the literature, we found it rich. However, due to constraints on article length and research focus, this paper primarily emphasizes the mechanisms of action of these drugs and their metabolites in obesity. Consequently, numerous aspects of translational pharmacology could not be elaborated upon in detail, warranting further in-depth exploration in future studies.
