# Supplementary material for: Gut microbiota and spleen-strengthening and dampness-dispelling therapies in obesity and related metabolic disorders: key mechanisms and therapeutic potential
Source: Front Pharmacol. 2026 Mar 11;17:1674533. doi: 10.3389/fphar.2026.1674533 (PMC13013495; doi:10.3389/fphar.2026.1674533)
Supplement: Supplementary file 2 [file Supplementaryfile1.pdf]

Supplementary Material

Supplementary Material

1 Figures

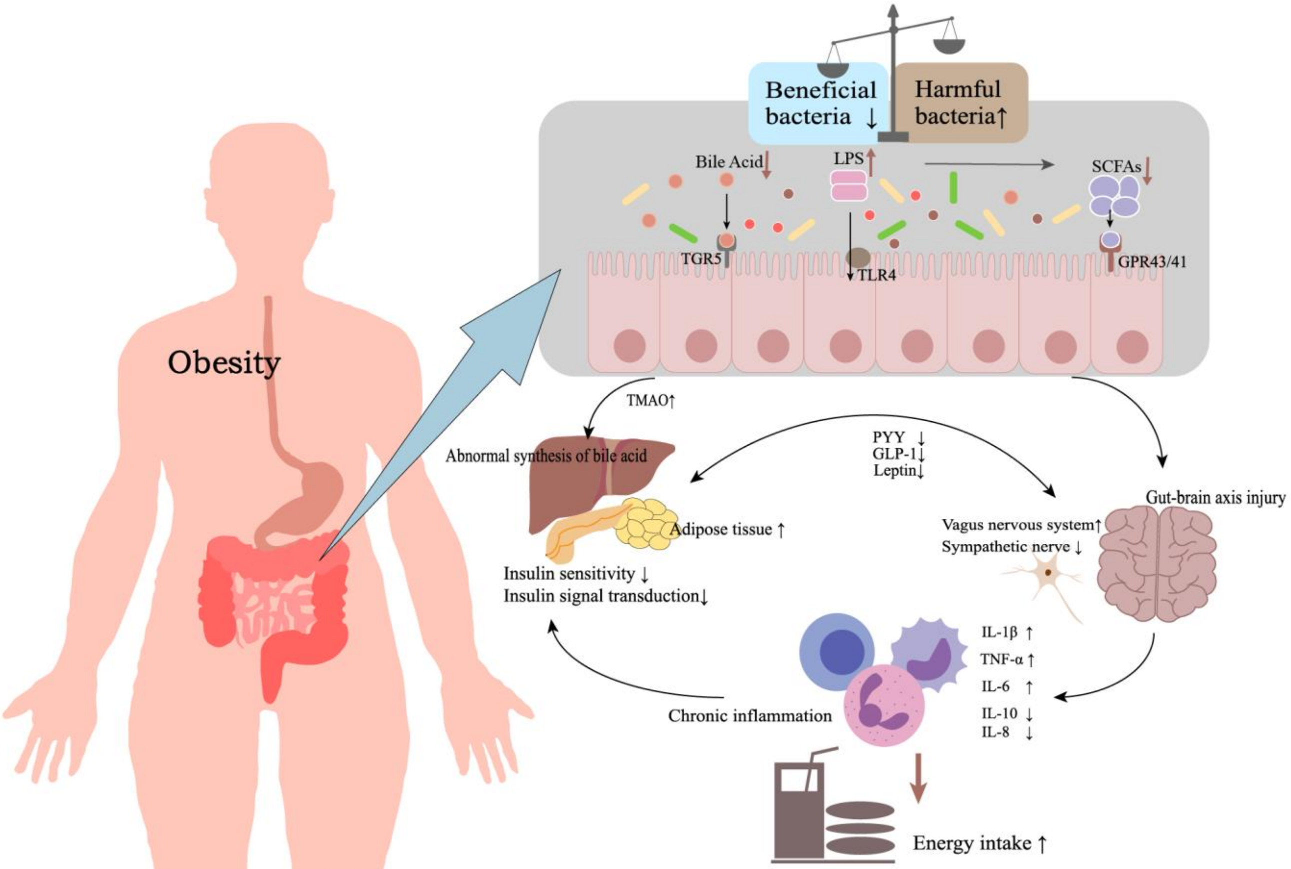

Figure 1: The Role of Gut Microbiota in Obesity Development. **Notes:** Obesity is an imbalance of energy metabolism caused by chronic inflammation, brain-gut axis damage, and abnormal bile acid metabolism, and is related to the gut microbiota and its related metabolic products. **Abbreviations:**TGR5, G protein-coupled bile acid receptor-5; TLR4, Toll-like receptor-4; LPS, lipopolysaccharide; SCFAs, short-chain fatty acids; GPR43/41, G protein-coupled receptors 43/41; TMAO, trimethylamine-N-oxide; PYY, peptide YY; GLP-1, glucagon-like peptide 1; TNF-α, tumor necrosis factor-α; IL-6, interleukin-6; IL-10, interleukin-10; IL-8, interleukin-8; IL-1β, interleukin-1β.

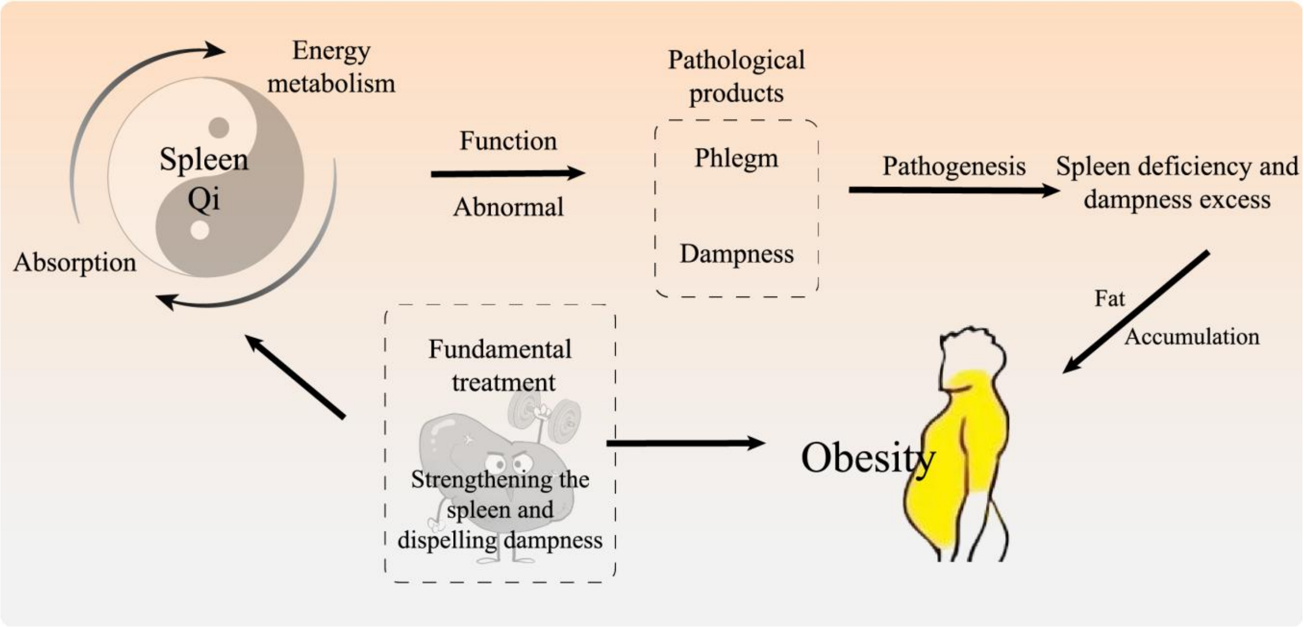

Figure 2: The Occurrence of Obesity under the Understanding of TCM. **Notes:** TCM believes that spleen deficiency and dampness excess caused by deficiency of spleen qi is the key to obesity. Phlegm and dampness are the pathological product, which in turn affects the function of the spleen. Therefore, the principle of treatment is to strengthening the spleen and dispelling dampness.

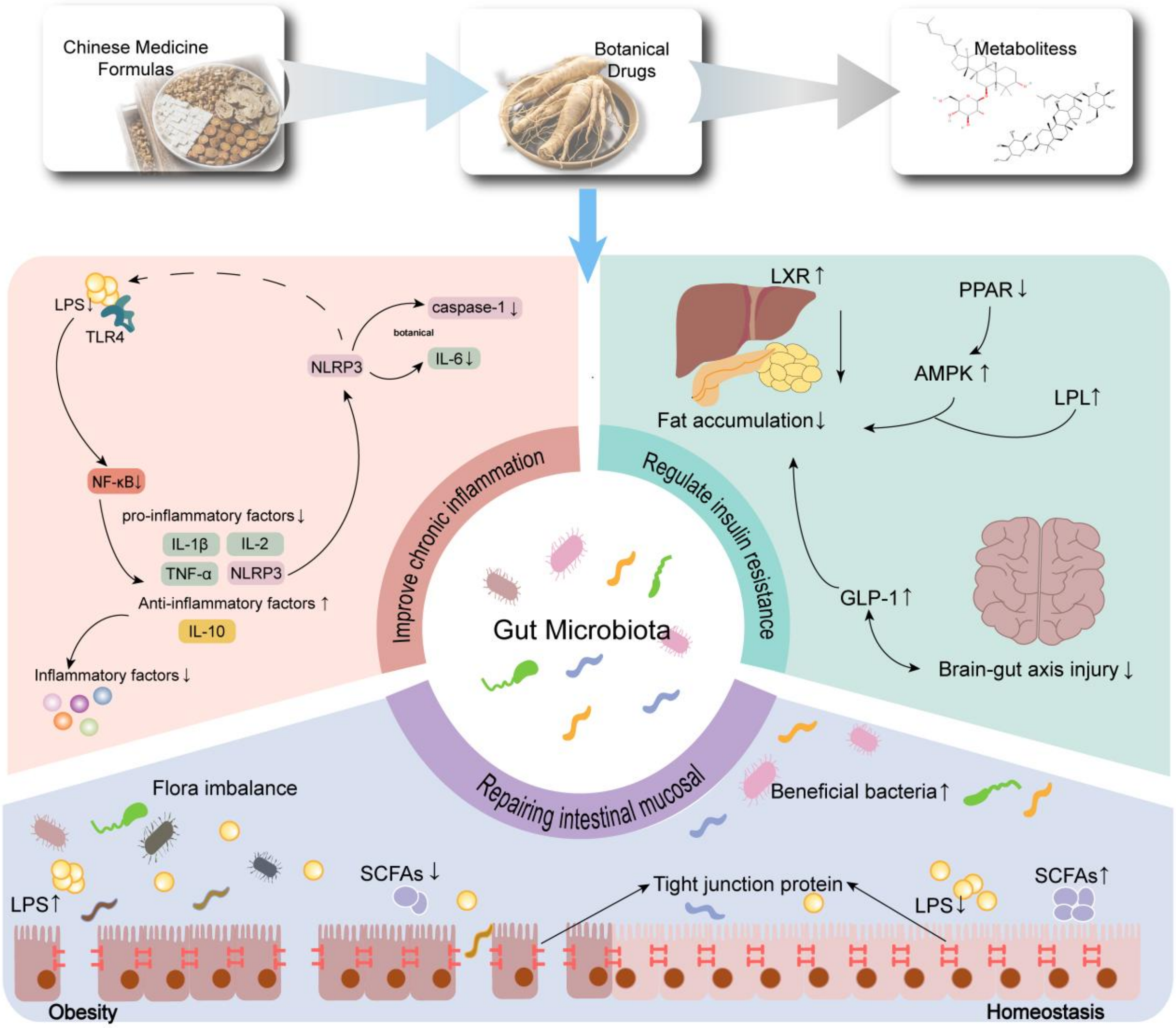

Figure 1: Common Potential Mechanism of TCM formulas and botanical drugs that strengthen the spleen and dispel dampness in improving obesity. **Notes:** The common mechanism involves maintaining gut barrier function, reducing chronic inflammation, and enhancing insulin-regulated energy metabolism. **Abbreviations:** NF-κB in the figure refers to the NF-κB pathway; NLRP3 in the figure refers to the NLRP3/caspase-1 pathway; AMPK: in the figure refers to the AMPK pathway. TLR4: Toll-like receptor-4; LPS: lipopolysaccharide; SCFAs: short-chain fatty acids; GLP-1: glucagon-like peptide 1; TNF-α: tumor necrosis factor-α; IL-6: interleukin-6; IL-2: interleukin-2; IL-10: interleukin-10; IL-1β: interleukin-1β; LXR: liver X receptor; PPAR γ: peroxisome proliferator-activated receptor γ; LPL: lipoprotein lipase.

2 Tables

Table 1 Summary of the study of gut microbiota and SCFAs on energy metabolism

Study	Design	Measurement	Gut microbiota	Metabolites related to microbiota	Associated factors
SATO et al.	sPLA2-X-deficient (Pla2g10-/-) mice	A high-fat diet (60% fat calories)	*Lachnospiraceae NK4A136 group*↓, *Lachnoclostridium*↓, *Roseburia and Turicibacter*↓	SCFAs↓	adipocyte hypertrophy↑<br>Th1, Tc1↑<br>alanine aminotransferase (ALT)↑<br>genes related to protection of the epithelial barrier↓<br>inflammatory genes↑<br>ω3 PUFAs ↓
Kulkarni et al.	C57BL/6 mice	antibiotic depletion，Fecal bacteria transplantation (FMT)(from obese patients)	*Colidextribacter*↓, *Faecalibaculum*↓, *Lachnospiracea*↓, *Oscillibacter*↓, *Phascolarcctobacterium*↓, *and Rosburia*↓	-	IL-6, MCP-1 and IL-1β↑<br>IL-17 and IFNγ↑<br>genes related to lipid uptake and metabolism↑

Fredrik et al.	Fiaf−/−mice	A high-fat diet (irradiated Western diet)	*Bacteroides thetaiotaomicron*↑ *and Methanobrevibacter smithii*↑	-	triglyceride↑<br>phospho-AMPK↓<br>serum glucose and insulin↓
Fredrik et al.	Rag1-/- mice	germ-free(GF) colonization，FMT (*Bacteroides thetaiotaomicron* strain VPI-5482)	-	-	total body fat content↑<br>metabolic rate↓<br>Leptin↑<br>liver triglyceride↑<br>acetyl-CoA carboxylase (Acc1), and fatty acid synthase (Fas)↑
Lu et al.	C57BL/6 J male mice	A high-fat diet (60% fat calories, mix with SCFAs or not)	*Firmicutes*↑*, Bacteroidetes*↓*,* Protein bacteria↑, *Actinobacteria*↓, *Candidate_division_TM7*↓, *Ruminococcaceae*↑*, Lachnospiraceae*↑*,*	-	glucose, triglycerides, cholesterol and insulin↑<br>free fatty acids↓<br>IL-1β, IL-6, IL-10 and MCP-1 ↑

			Anaerotruncus↑ *and Lactobacillus*↑		GPR41 and GPR43↑<br>GLP-1 and PYY↑<br>cpt1a, cpt1c and cpt2↓<br>the genes involved in mitochondrial biogenesis↓
Samuel et al.	Gpr41−/− mice	FMT(B. *thetaiotaomicron* strain VPI-5482 and M. *smithii* strain PS)	-	SCFAs↑	GPR41, GPR43, and GPR120 mRNAs↓<br>fat pat weight↑<br>serum leptin↓<br>Fas↓
Chambers et al.	Randomized Controlled Trial	10 g/day inulin-propionate ester	*Bifidobacterium spp*↑*, Atopobium cluster*↑*, Bacteroides/Prevotella* ↑	-	GLP-1 and PYY↑<br>subcutaneous adipose tissue↓<br>Aspartate transaminase (AST) and ALT↓

Lin et al.	Free fatty acid receptors 3-deficient mice	A high-fat diet with SCFAs	-	-	leptin↓<br>insulin↓<br>GLP-1, PYY, and amylin↑<br>plasma ghrelin↓<br>GIP↑
Hong et al.	C57BL/6 J male mice	A high-fat diet (40% fat calories), sodium butyrate in drinking water	-	-	the epididymal fat mass↓<br>glucose, insulin and leptin↓<br>triglyceride and total cholesterol↓<br>AMP, ATP and ADP↑<br>hormone sensitive lipase (HSL) and lipoprotein lipase (LPL) ↑<br>the genes involved in mitochondrial thermogenesis↑

the genes involved in fatty acid β-oxidation↑

phospho-AMPK↑

histone deacetylase 1(HDAC1)↓

Table 2 Summary of the study of gut microbiota on response of host inflammation

Study	Design	Measurement	Gut microbiota	Metabolites related to microbiota	Associated factors
Macia et al.	GPR43−/− and GPR109A−/− mice	high fibre diet, sodium acetate in drinking water	*Bacteroidiaceae*↓, *TM7* and *Oscillibacter* ↓, *Prevotellaceae*↑, *Lachnospiraceae*↑, *Alcaligenaceae*↑ and *Helicobacteraceae*↑	-	IL-18↑
Gaudino et al.	IL22−/− mice	A high-fat diet	*Oscillibacter*↑	-	not affect expression of IL-1β<br><br>ileal lipid metabolism genes↑
Virtue et al.	miR-181-deficient mice (DKO)	A high-fat diet, antibiotic depletion, FMT(*tryptophanase-sufficient* or *tryptophanase-deficient* strains of *E. coli.*)	-	indole-3-carboxylic acid and indoxyl sulfate↓	lipid droplet deposition↑<br><br>lipid catabolizing (Acox1 and Lipe) genes↑<br><br>glucose clearance↓<br><br>triglycerides↑

					Ppara, Lipe, and Pnpla2(triglyceride hydrolyzer)↓<br><br>epididymal white adipose tissue↑<br><br>O2 consumption, CO2 production ↑<br><br>insulin↓, insulin resistance↑<br><br>Tregs, eosinophils, and ILC2s↑<br><br>M2 macrophage polarization genes↑<br><br>pro-inflammatory M1 macrophage polarization genes↓
Cani et al.	CD14 mutant mice	A high-fat diet(72% fat), infusion of LPS	*Cytophaga*↓, *Eubacterium rectale-Clostridium coccoides group*↓ and *Bifidobacteria*↓	-	fasting blood-glucose↑
Jain et al.			*Proteobacteria*↑	-	fasted insulin↑

	C57BL/6 male mice	A high-fat diet (60% fat calories)			TNF-α, IL-1, IL-6, and PAI-1 mRNA↑<br><br>LPS on the surface of faecal-EVtotal↑<br><br>TLR4↑
Mckernan et al.	TLR4-/- male mice	A diet with 4.5% fat, Mouse bone marrow cells were cultured(LPS or Palmitate treatment)	-	-	F4/80, CD86 and CD206↑<br><br>inflammatory responses genes↑<br><br>lipid↑<br><br>Pparγ, Cebpα, and Pgc1α(genes)↓<br><br>TNF-α，IL-6 and MCP1↑

Table 3 Summary of the study of gut microbiota participates in bile acid metabolism

Study	Design	Measurement	Gut microbiota	Metabolites related to microbiota	Associated factors

				BAs profiles in portal↑	
Adyin et al.	cohort study	the ages of ≥18 and ≤64 y<br>Body mass index of ≥ 35 kg/m2	*Actinomycetota*↑, *Bacillota*↑, *Bacteroidota*↑ and *Pseudomonadota*↑	diversity of BAs in portal↓(Glycocholic acid (GCA), glycodeoxycholic acid (GCDCA), glycerodeoxycholic acid (GDCA), taurocholic acid (TCA), taurochenodeoxycholic acid (TCDCA), taurodeoxycholic acid (TDCA))	FGF19 in portal↑
Sayin et al.	FXR−/− mice	antibiotic depletion, GF feeding, TCA or a mixture of TCA and (tauro-β-muricholic acid)TβMCA	-	BAs↑, taurine↑<br>TβMCA↑, TCA and Tauro-α-muricholic acid(TαMCA)↓	cholesterol and phospholipids↓(GF mice)<br>CYP7A1↑(GF mice)<br>bile acid transporters genes↑(GF mice)<br>molecular targets Shp and Fgf15↓<br>FXR↓
			Bacteroidia↓,	-	fasting glucose↑

Ava et al.	FXR−/− germ-free mice	GF feeding, FMT(from FXR−/− mice with high-diet)	*Firmicutes*↑		oral glucose tolerance↓<br>macrophage markers Emr1 (encodes the protein F4/80)↑<br>Saa3 and Tnfa (encode proinflammatory cytokines) ↑<br>Ccl2 (promotes macrophage infiltration into WAT)↑<br>triglycerides, saturated triglycerides and cholesteryl esters↑<br>fatty acid transporter Cd36, Apoc2 and Vldlr↓
Zheng et al.	C57BL/6 male mice	a high-fat diet supplemented with GW4064 (FXR agonist)	*Firmicutes*↑, *Proteobacteria*↑, and *Actinobacteria*↑; *Verrucomicrobia*↓, *Bacteroidetes*↓ and *TM7*↓; *Ruminococcus gnavus*↑, *Blautia spp.*↑, *Oscillospira*	total BAs, cholic acid(CA),GCA,TDCA,TCA, chenodeoxycholic acid (CDCA), deoxycholic acid (DCA),TCDCA, β-muricholic acid (βMCA), lithocholic acid (LCA), and 7-ketolithocholic acid (7-ketoLCA)↑, β-cholic acid (βCA)↓	bady weight↑

			spp.↑, and *Bilophila spp.*↑		
Wang et al.	Gpbar1 (known as TGR5) global knock out(KO) male mice	antibiotic depletion,GF colonization（C57BL/6J donor mice）	-	total BAs↑, TCA and TβMCA↓	GLP-1↓<br>TGR5↓
Mo et al.	SD male rat	A high-fat diet (45% fat calories), TMAO in drinking water	*Firmicutes*↑, *Mucispirllum*↑; *Bacteroidetes*↓, *Flavonifractor*↓ and *Intestinimonas*↓	SCFAs↓	muscles mass↓<br>lipid↑<br>Muc-2 and goblet cells↓
Tan et al.	C57BL/6J male mice	A high-fat diet (45% fat	-	TCA↑, DCA and TDCA↓	AST↑<br>total cholesterol↑

calories),TMAO in drinking water		hepatic lipogenic genes Srebp-1c and Fas↑<br><br>medium-chain acyl-CoA dehydrogenase (Mcad) mRNA↓<br><br>microsomal triglyceride transport protein (Mtp) mRNA↓<br><br>CYP7A1 mRNA↑<br><br>FXR mRNA↑, FXR↓

Table 4 Summary of the study of gut microbiota involved in the gut-brain axis

Study	Design	Measurement	Gut microbiota	Metabolites related to microbiota	Associated factors
Liu et al.	female C57BL/6J mice and its offspring	A high-fat diet	*S24-7*↓, *Bifidobacterium animalis*↓, *Prevotella*↓, and *Clostridiales*↓	acetate and propionate↓	three-chamber sociability test, the Y-maze test,PSD-95, FXR1, FXR2, TDP2, GluN2B, and GluA2↓; MAFB, BDNF, NGF, DAP12, and CX3CL1↓, CD31 and F4/1↑
Frost et al.	C57BL/6J male mice	A high-fat diet (41.8% energy from fat), supplemented with inulin or cellulose	-	total SCFA, acetate↑	signal intensity in the arcuate nucleus↑, AMPK↓,γ-amino butyric acid (GABA), lactate(Lac), glutamate(Glu)↑
Kaneko et al.	C57BL/6J mice	A high-fat diet (60% calories fat), Gipg013(GIP receptor antagonist) brain injection	-	-	glucose, leptin and insulin↓,STAT3↓, GIP ↑,Rasrelatedprotein 1 (Rap1)↑

Table 5 Comparison table of TCM concepts and indicators

TCM Concepts	Biomedical Manifestations
Spleen Qi Deficiency	gastric (GAS)
	motilin (MTL)
	somatostatin (SS)
	CD4/CD8
	ATP
	AMPK
	MMP
	gastric emptying
	small intestinal propulsion
	5-HT
	visceral sensitivity

	Treg cells
	D-xylose content
	IFN-γ
	IL-2
	T-CHO
	ROS
	IL-6
Dampness excess	TNF-α
	IL-17
	IL-10
	TG
	HDL

LDL

SOD

MDA

Table 6 Summary of pre-clinical studies of TCM formulas

Formulas	Intervention	Model	Dosages	Target/Factors	Mechanism	Gut microbiota	Literatures
Sijunzi decoction							
	S-3-1(purified from Sijunzi decoction)	in vitro	2 mg/mL, 1 mg/mL	Acetic acid↑, Propionic acid↓, Butyric acid↓	Regulation of gut microbiota and SCFAs	*Bacteroidetes*↑, *Firmicutes*↓, *Oscillospira*↑, *Streptococcus*↓, *Enterococcus*↓, *Clostridium*↑, *Bacteroides*↓, *Dorea*↑, *Lactobacillus*↓, *Pediococcus*↓	Gao et al.(2018)
	Standardized Sijunzi decoction extract	Spleen deficiency syndrome rats, antibiotic depletion	10 g/kg·d, 7 days	gastrin (GAS)↓, motilin (MTL)↓, somatostatin (SS)↓, Ghrelin (GRHL)↓, interleukin-2 (IL-2)↓, interferon-gamma (IFN-γ)↓,CD4/CD8 ratio↑	the regulation of GI hormones, Immunomodulatory effect, modulation of the gut microbiota composition	*Actinobacteria*↑, *Rothia*↑, *SMB53*↑, *Lactobacillus*↓, *Collinsella*↑, *Butyricimonas*↓	Ma et al.(2021)
	Sijunzi decoction	Spleen Qi	1.4g/d, 2.8g/d,	ATP↑, MMP↑, reactive oxygen species (ROS)↓, microtubule-associated protein light chain 3 (LC3)↑,	Inhibition of mitochondrial autophagy	-	Liu et al.(2021)

	deficiency rats	5.6g/d, 14 days	sequestosome 1 (p62)↓, AMPK↓,unc-51 like autophagy activating kinase 1 (ULK1)↓,			
Xiangsha Liujunzi decoction concentrated dry powder	in vitro, functional dyspepsia rats	0.36 g/ml, 0.18 g/ml, 0.09 g/ml,14 days	gastric emptying↑, small intestinal propulsion↑, autophagosomes↓, LC3↑, p62↓, PINK1↓, mitochondrial reactive oxygen species(mtROS)↓	Improvement of mitochondrial function	-	Zhang et al.(2022)
Xiangsha Liujunzi decoction	Functional dyspepsia rats	1ml/100g, 7 days	incremental balloon pressure↓, number of EC cells↓, paired box gene 4 (PAX4)↑,5-Hydroxytryptamine (5-HT)↓, tryptophan hydroxylase-1 (TPH1)↓,5-hydroxytryptamine 3 receptor (5-HT3r)↓	Decrease of visceral sensitivity	-	Zhao et al.(2020)
Xiangsha Liujunzi decoction	Helicobacter pylori-related gastritis rats	15ml/kg, 4 weeks	tumor necrosis factor-alpha (TNF-α)↓， IL-6↓, inducible nitric oxide synthase (iNOS)↓, nitric oxide (NO)↓,mitogen-activated protein kinase (MAPK)↓,	Reduction of inflammation	-	Lin et al.(2016)

				nuclear factor kappa-B (NF-κB)↓,TLR2↓, TLR4↓			
Shenling Baizhu powder	Concentrated solution of Shenling Baizhu powder	Rotavirus enteritis rats, in virtro	8.37 g/kg, 3 days	TLR4↓,Myeloid Differentiation Primary Response Gene 88 (MyD88)↓, NF-κB↓, IL-1β↓, IL-6↓, TNF-α↓, IFN-β ↑	Alleviation of inflammation	-	Wang et al.(2021)
	Shenling Baizhu Powder	Spleen Qi deficiency rats	0.93, 1.86, and 3.72 g/kg, 7 days	D-xylose content↑, IL-10↑, IL-17↓, Treg cells↑	Regulation of intestinal function and immunity	-	Xiao et al.(2021)
	Shenling Baizhu decoction	Mouse model of diarrhea caused by lard	0.25g/d, 14 days	MDA↓, SOD↑, LDL↓, TG↓, SS↓, CCK↓, IL-17↓, IL-6↓， SCFAs↑	Improvement of fat metabolism and inflammation	*Lactobacillus reuteri*↑, *Lactobacillus intestinalis*↑	Qiao et al.(2024)
	Shenling Baizhu decoction	Pelotinib-induced diarrhea rats	3.6 g/kg, 10 days	mucosal damage(pathological change)↓, cyclic AMP(cAMP)↑, 25-hydroxycholesterol↑,	Changes of gut microbiota and its metabolites	*Lachnospiraceae*↑, *Bacilli*↑, *Lactobacillales*↑, *Allobaculum*_	Lai et al.(2022)

				guanidinosuccinic acid↑, 5-hydroxyindolepyruvate↑		*stercoricanis*↑, opportunistic bacteria↓	
	Shenling Baizhu decoction	High-fat diet rats	0.75g/d, 16 weeks	ALT↓, AST↓, T-CHO↓, TG↓, IL-1β↓, IL-18↓, TNF-α↓, LPS↓,TLR4↓,NLRP3↓,MyD88↓,caspase 1↓	Improvement of fat metabolism and inflammation	*Bacteroidetes*/*Firmicutes* ratio↑, *Bifidobacterium*↑, *Akkermansia*↓	Zhang et al.(2018)
	Shenling Baizhu decoction	Spontaneously obese type 2 diabetic rats	0.66g/d, 0.132g/d, 0.264g/d, 14 weeks	glycated hemoglobin(Hb1Ac)↓, TG↓, T-CHO↓, LDL↓, HDL↓, glucose↓,	Improvement of fat metabolism, Changes of gut microbiota	*Prevotella*↑, *Anaerostipes*↑, *Turicibacter*↑, *Bilophila*↑, *Ochrobactrum*↑, *Acinetobacter*↑, *Lactobacillus*↓, *Roseburia*↓, *Staphylococcusc*↓	Zhang et al.(2021)
Erchen Decoction	Erchen Decoction	High-fat diet mouse	8.7g/kg·d, 14 weeks	TG↓, T-CHO↓, PPARγ↑, LPL↑	Improvement of fat metabolism	-	Zhang et al.(2020)
	Concentrated solution of Erchen Decoction	Zucker diabetic fatty rats	2.28 g/kg, 4.57 g/kg, 9.14 g/kg, 5 weeks	ITT↓, IRS1↓, AKT↑, PKA↓, HSL↓,TC, HDL↓, LDL-C↓, TG↓	Improvement of fat metabolism, Changes of gut microbiota	*Prevotella*↓, *Ruminococcus*↓, *Blautia*↓, *Holdemania*↓	Zhao et al.(2021)

Erchen Decoction	High-fat diet rats	2.28 g/kg, 4.57 g/kg, 9.14 g/kg, 4 weeks	TC↓, TG↓, LDL↓, HDL↓, FFA↓, AST↓, ALT↓, butyric acid↑, HDAC1↓, H3K9ac↑	Improvement of fat metabolism, Changes of gut microbiota	*Butyricicoccus*↓, *Bifidobacterium*↓, *Lactobacillus*↓, *Coprobacillus*↑, *Eubacterium*↓	Zhang et al.(2023)
Erchen Decoction	High-fat diet mouse	5.7 g/kg, 6 weeks	ALT↓, AST↓, LPS↓, TLR4↓, TNF-α↓, IL-1β↓, NF-κB↓, TG↓, TC↓, FFA↓, tight junction proteins (claudin-3, occludin, and ZO-1)↑, acetic acid↑, propionic acid↑, butyric acid↑	Improvement of fat metabolism and inflammation, Changes of gut microbiota	*Firmicutes*↑, *Proteobacteria*↑, *Bacteroidetes*↓, *Cyanobacteria*↓, *Verrucomicrobia*↓	Liu et al.(2021)
Erchen Decoction	High-fat diet rats	4.5 g/kg, 9 g/kg, 12 weeks	SOD↑, glutathione peroxidase (GSH-Px)↑, MDA↓, IL-6↓, IL-1β↓, TNF-α↓, ALT↓, AST↓, TG↓, T-CHO↓	Improvement of fat metabolism and inflammation, Changes of gut microbiota	*Bacteroidetes*/*Firmicutes* ratio↑, *Lactobacillus*↑, *Dubosiella*↑, *Lachnospiraceae*↑, *Akkermansia*↑, *Intestinimonas*↑, *Desulfovibrio*↓, *C._saccharimonas*↓	Miao et al.(2022)

Daotan Decoction	Cangfu Daotan Decoction	obese PCOS model rats	1.42 g/kg, 5.68 g/kg, 2 weeks	insulin↑, T-CHO↓, testosterone (T)↓, luteinizing hormone (LH)↓, TG↓, LDL↓, HDL↑, stimulating growth hormone (FSH)↑, estradiol (E2)↑, IL-2↓, IL-6↓, TNF-α↓, OATP3A1↑, OATP2B1↑	Improvement of fat metabolism and inflammation, Regulation of hormone level	-	Yi et al.(2021)
	Huanglian Wendan Decoction	High temperature, high humidity environment, high fat diet rats	7.8g/kg, 4 weeks	NF-κB↓, NLRP3↓, caspase-1↓, IL-1β↓, IL-18↓, ISI↑, IRI↓, 2-hour postprandial glucose (2hPG)↓	Improvement of inflammation and glycometabolism	-	Dong et al.(2021)
	Huanglian Wendan Decoction	Diabetic encephalopathy rats	3g/kg, 6g/kg, 30 days	Fasting blood-glucose (FBG)↓, TG↓, T-CHO↓, TNF-α↓, IL-6↓, IL-1β↓, neuron density↑, amyloid deposition↓, AKT↑, IRS-1↑	Improvement of inflammation, fat metabolism and glycometabolism	-	Li et al.(2016)

Table 7 Summary of clinical studies of TCM formulas

Formulas	Studies	Intervention	Duration	Participants	Key Microbiome	Key Factors	Literatures
Sijunzi decoction	Observational with Intervention (for the treatment group)	Sijunzi decoction	12 weeks	Healthy group, Patients with chronic atrophic gastritis (CAG) (CAG group), Treatment group	*Erysipelotrichia*↓, *Bacteroides*↓, *Blautia*↓, *Faecalibacterium*↑, *Fusicatenibacter*↑	-	Xing et al.(2024)
Shenling Baizhu powder	RCT	Shenling Baizhu powder (12 g × 10 bags/box), 18g/d; Mesalazine enteric-coated tablets (0.25 g)	30 days	patients (aged 60-78 years) with ulcerative colitis complicated with bloody purulent stool	-	bloody purulent stool↓, IL-2↓, IFN-γ↓	Li et al.(2021)

Table 8 Summary of pre-clinical studies of botanical drugs

Botanical Drugs	Intervention	Model	Dosages	Target/Factors	Mechanism	Gut microbiota	Literatures
Ginseng							
	Ginseng extracts	In vitro	0.4 mg/mL, 48 hours	-	Changes of gut microbiota	*Streptococcus*↓, *Escherichia-Shigella*↓, *Veillonella*↓, *Lactobacillus*↓, *Bifidobacterium*↓, *Enterococcus*↓, *Actinobacteria*↓, *Bacteroidetes*↑	Li et al.(2020)
	Ginseng extracts	High-fat diet mouse, FMT, antibiotic depletion	10 mg/kg, 2 weeks	Brown adipose tissue (BAT)↑, UCP1↑, OXPHOS↑	Improvement of fat metabolism, Changes of gut microbiota	*Enterococcus*(Genus) ↑, *Enterococcus faecalis*(Species)↑	Quan et al.(2020)
	Red ginseng extracts	spleen qi deficiency rats	3.24 g/kg, 6.48 g/kg, 10 days	D-xylose↑, VIP↓, SP↑, AChE↑, ACTH↑, CORT↑, T3↑, T4↑, E2↑, 5-HT↑, CS↑, NCR↑, IDH1↑, COX↑, Na+-K+-ATPase↑, cAMP↑, cGMP↓, acetic acid↓, propionic acid↓,	Changes of gut microbiota, Regulation of GBA and energy metabolism	*Bacteroidetes*/*Firmicutes*↑, *Lactobacillus*↑, *Akkermansia*↓	Zhang et al.(2023)

				isobutyric acid↑, butyric acid↑, isovaleric acid↑			
	Protopanaxadiol ginsenosides	In vitro	15 mg/mL, 24 hours	-	Changes of gut microbiota	*Prevotella_9*↑, *Faecalibacterium*↑, *Dialister*↑, *Escherichia-Shigella*↓, *Dorea*↓, *Lachnoclostridium*↓	Zhang et al.(2021)
Atractylodes macrocephala Koidz	Atractylodes macrocephala Koidz	Ferroptosis goslings	400 mg/kg, 28 days	Ferroptosis genes (GPX4, FTH1, FPN1, HSPB1, COX-2, NOX1, TFR1, ACSL4)↓, IFN-γ↓, IL-1β↓, IL-4↓, IL-6↓, IL-10↓, IL-17↓, IL-18↓, TNF-α↓	Improvement of ferroptosis and inflammation	-	Li et al.(2022)
	Atractylodes macrocephala Koidz	DSS-induced colitis mouse	100mg/kg, 14 days	ZO-1↑, claudin↑	Changes of gut microbiota	*Bacteroides*↑, *Lactobacillus*↑	Zhang et al.(2025)
	Atractylodes macrocephala Koidz	DSS-induced colitis mouse	100mg/kg, 200mg/kg, 400mg/kg, 15 days	IL-1β↓, IL-6↓, TNF-α↓, ZO-1↑, claudin-1↑, Occludin↑, MUC-2↑	Improvement of inflammation,	*Clostridium sensu stricto 1*↓, *Escherichia Shigella*↓	Kai et al.(2022)

					Changes of gut microbiota		
Chinese yam	Chinese yam ethanol extract	SD rats	Add 2% or 10% to food, 6 weeks	neutral lipids↓	Changes of gut microbiota	*Bacteroides fragilis*↓	Jeon et al.(2006)
	Chinese yam extract	In vitro	24 hours	acetic acid↑, butyric acid↑	Changes of gut microbiota	*Clostridium*↑, *Lactobacillus*↑, *Akkermansia*↑	Cui et al.(2023)
	Chinese yam	Antibiotic-associated diarrhea mouse	30 mg/kg, 14 days	IL-1β↓, IL-6↓	Improvement of inflammation, Changes of gut microbiota	*Bacteroides thetaiotaomicron*↑, *Paramuribaculum intestinale*↑	Pan et al.(2022)
Poria cocos	Water insoluble polysaccharid e	ob/ob mice	1g/kg, 0.5g/kg, 4 weeks	T-CHO↓, TG↓, LDL↓, SOD↑, TNF-α↓, LPS↓, ISI↑, glucose↓, insulin↓, AST↓, ALT↓	Improvement of fat metabolism and inflammation, Changes of gut microbiota	*Lachnospiracea*↑, *Alloprevotella*↑, *Parabacteroides*↑, *Clostridum IV*↑, *Ruminococcus*↑, *Bacteroides*↑, *Megamonas*↓, *Proteus*↓	Sun et al.(2019)

	Poria cocos oligosaccharides powder	High-fat diet mouse, FMT, antibiotic depletion	200mg/kg, 8 weeks	Glucose tolerance test↓, insulin tolerance test↓, insulin↓, TNF-α↓, IL-1β↓, IL-6↓, COX-5b↓, MCP-1↓, GPR43↓, NLRP3↓, FXR↓, FGF15↓,CA↑, UDCA↑, valeric acid↓, 5-HT↓	Improvement of fat metabolism and inflammation, Changes of gut microbiota	*Bacteroidetes/Firmicutes*↑, *Ruminococcaceae*↓, *Anaeroplasmataceae* ↓, *Lactobacillaceae*↑, *Rikenellaceae* ↑	Zhu et al.(2022)
Glycyrrhiza	Glycyrrhiza extract	Type 2 diabetes mice	20mg/kg, 40mg/kg, 80mg/kg, 4 weeks	Insulin↓, LPS↓, insulin resistance↓, HDL↑, TG↓, LDL↓, T-CHO↓, ALT↓, AST↓, occludin↑, ZO-1↑, IL-6↓, IL-12↓, TNF-α↓, NF-κB↓, TLR4↓, IKKα↓	Improvement of fat metabolism and inflammation, Changes of gut microbiota	*Bacteroidetes*↑, *Firmicutes*↓, *Alloprevotella*↑, *Bacteroides*↑, *Lachnospiraceae_NK4A136_group*↓, *Akkermansia*↑	Zhang et al.(2022)
	Glycyrrhiza extract	High-fat diet mouse, antibiotic depletion, in vitro	0.2 mL, 4 weeks	AST↓, alkaline phosphatase (ALP)↓, cholinesterase (CHE)↓, TG↓, T-CHO↓, LDL↓, TNF-α↓, IL-6↓, IL-10↑, fatty acid synthase (FASN)↑, acylglycerol lipase (MGL)↑, SCFAs↓	Improvement of fat metabolism, Changes of gut microbiota	*Clostridium sensu stricto 1*↓, *Lactobacillus*↓	Liu et al.(2022)

Rhizoma Alismatis	Rhizoma Alismatis water extract	High-fat and high-sugar diet rats	2.1g/kg, 14 days	T-CHO↓, LDL↓,	Improvement of fat metabolism and inflammation, Changes of gut microbiota	*Lactobacillus*↓	Xu et al.(2020)
Pericarpium Citri Reticulatae	Pericarpium Citri Reticulatae extract	High-fat diet mouse	5 g/kg, 10 g/kg, 12 weeks	T-CHO↓, TG↓, HDL↑, LDL↓	Improvement of fat metabolism and inflammation, Changes of gut microbiota	*Firmicutes*↓, *Bacteroides*↑, *Anaerotruncus*↑, *Odoribacter*↑, *Rikenellaceae_RC9_gut_group*↑, *Alistipes*↑, *Ruminiclostridium_9*↑	Li et al.(2021)

Table 9 Summary of pre-clinical studies of metabolites

Botanical Drug	Metabolites	Structural formulas	Intervention	Model	Dosages	Target/Factors	Mechanism	Gut microbiota	Literatures
Ginseng				High-fat diet mouse	30mg/kg, 60mg/kg, 7 weeks	IL-6↓, TNF-α↓, IL-1β↓, LPS↓, glucose tolerance↓, ZO-1↑, occludin↑, claudin↑, TLR4↓, MYD88↓, NF-κB↓, IκB-α↑, total SCFAs↑	Improvement of inflammation, Changes of gut microbiota	*Bacteroidetes/Firmicutes*↑, *Actinomycetes*↑, *Bifidobacteria*↑, *Lactobacilli*↑	Chen et al.(2021)
	Ginsenoside Rk3	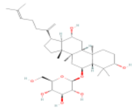	Ginsenoside Rk3	Antibiotic-induced gut microbiota dysbiosis mouse	20mg/kg, 60mg/kg, 2 weeks	IL-6↓, TNF-α↓, IL-1β↓, IL-17↓, IL-10↑, IFN-γ↑, ZO-1↑, occludin↑, claudin↑,acetate↑, propionate↑, butyrate↑	Improvement of inflammation, Changes of gut microbiota	*Bacteroidetes/Firmicutes*↑	Bai et al.(2021)
				High-fat diet mouse	30mg/kg, 60mg/kg, 8 weeks	MDA↓, SOD↑, LDL↓, HDL↑, TG↓, T-CHO↓, PGE2↓, PGD2↓,	Improvement of inflammation, Changes of	-	Wang et al.(2024)

					TXB2↓, HETE↓, HODE↓	gut microbiota		
Ginsenoside F2	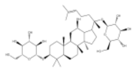	Ginsenoside F2 purified from yeast	Wild type and LXRα deficient mouse with a high-fat diet	50mg/kg, 12 weeks	ALT↓, AST↓, Srebf1↓, Fasn↓, Il1b↓, Tnf↓, Il6↓	Improvement of fat metabolism and inflammation	-	Kim et al.(2024)
		Ginsenoside F2	In vitro	-	AMPK↑, ACC↓, T-CHO↓, TG↓, AST↓	Improvement of fat metabolism	-	Zhou et al.(2021)
Kaempferol	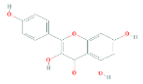	Kaempferol	Leptin receptor-deficient obese mice	50 mg/kg, 6 weeks	glucose tolerance test↓, insulin tolerance test↓, F4/80↓, TNF-α↓, IL-18↓, IL-10↓, NLRP3↓, caspase-1↓	Improvement of inflammation	-	Zhai et al.(2024)
		Kaempferol	High-fat diet mouse	High fat diet with 0.1%	T-CHO↓, TG↓, TNF-α↓, IL-1β↓, IL-	Improvement of inflammation	*Bacteroidetes/Firmicutes*↑, *Alistipes*↓,	Bian et al.(2022)

					kaempferol, 16 weeks	6↓,myeloperoxidase (MPO),TLR4↓, MyD88↓, NF-κB↓,F4/80↓	and fat metabolism, Changes of gut microbiota	*Lachnospiraceae_NK4A136_group*↓, *Romboutsia*↓, *Faecalibaculum*↓, *Kaempferol*↓	
			Kaempferol	High-fat diet mouse	200mg/kg, 8 weeks	TG↓, glucose↓, HDL↑, LDL↓, T-CHO↓	Improvement of fat metabolism, Changes of gut microbiota	*Firmicutes*↓, *Bacteroidetes*↑, *Proteobacteria*↑, *Akkermansia*↑	Wang et al.(2020)
Atractylodes macrocephala Koidz	Atractylenolide I	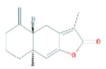	Atractylenolide I	Antibiotic-induced gut microbiota dysbiosis mouse, in vitro	-	LPS↓, IL-1β↓,TLR4↓, MyD88↓, NF-κB↓	Improvement of inflammation, Changes of gut microbiota	*Lactobacillus*↑, *Bacteroides*↑,*Escherichia*↓, *Candidatus*↓	Liu et al.(2021)
	Atractylenolide III	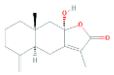	Atractylenolide III	TNBS-induced colitis moiuse	5mg/kg, 10mg/kg, 20mg/kg, 14 days	FPR1↓, DUOX2↓, NOX1↓, Nrf2↓, MDA↓, SOD↑,	Improvement of inflammation, Changes of	*Actinobacteria*↑, *Bacteroidetes*↑	Ren et al.(2021)

						GSH-Px↑, TNF-α↓, IL-1β↓	gut microbiota		
Chinese yam			Diosgenin	melanoma-bearing mouse, in vitro	20 mg/kg, 14days	CD4 cells↑, CD8 cells↑, IFN-γ↑	Immunoregulation，Changes of gut microbiota	*Lactobacillus*↑, *Sutterella*↑, *Bacteroides*↑	Dong et al.(2018)
	Diosgenin	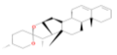	Diosgenin	DSS induced colitis mouse	15 mg/kg, 7days/14days	total SCFAs↑, acetic acid↑, propionic acid↑, isobutyric acid↑	Changes of gut microbiota	*Prevotella*↑, *Odoribacter*↑, *Mucispirillum*↑, *Veillonella*↑	He et al.(2022)
			Diosgenin	methionine and choline-deficient (MCD) feeding mouse, FMT	30 mg/10 mL/kg, 1 week	TG↓, T-CHO↓, ALT↓, AST↓, FXR↑, CYP7A1↓, fibroblast growth factor(FGF) 15↑	Improvement of BAs and fat metabolism, Changes of gut microbiota	*Clostridia*↑	Yan et al.(2023)
	Taxifolin	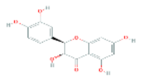	Taxifolin	High-fat diet mouse	0.5mg/mL, 1mg/mL, 15 weeks	SOD↑, TG↓, T-CHO↓, HDL↑,	Improvement of fat metabolism, Changes of	*Bacteroidetes*/*Firmicutes*↑, *Mucispirillum*↓,	Su et al.(2022)

						LDL↓,MDA↓, SOD↑,GSH-Px↓	gut microbiota	*Desulfovibrio spp*↓	
	Stigmasterol	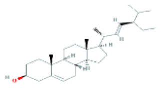	Stigmasterol	DSS induced colitis mouse, Antibiotic depletion, FMT	400 mg/kg, 10 days	IL-6↓, IL-1β↓, TNF-α↓, IL-10↑, IL-17A↓,acetate↑, propionate↑, butyrate↑, isobutyrate↑, valerate↑	Improvement of inflammation, Changes of gut microbiota	*Helicobacter*↑, *Odoribacter*↑, *Prevotella*↑, *Oscillospira*↑, *Paraprevotella*↑, *Turicibacter*↑, *Ruminococcus*↑, *Butyricicoccus*↑, *Ruminococcaceae*↑, *and Paraprevotellaceae*↑	Wen et al.(2021)
			Stigmasterol	High-fat and high-cholesterol diet mouse	200 mg/kg, 10 weeks	NLRP3↓, IL-18↓, T-CHO↓, TG↓, HDL↑, Caspase-1↓, IL-1β↓, CYP7B1↑	Improvement of inflammation, Changes of gut microbiota	*Lachnospiraceae_NK4A136_group*↓, *Desulfovibrio*↓, *Lactobacillus*↓	Xin et al.(2023)
Glycyrrhiza	Glabridin	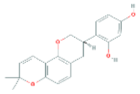	Glabridin	DOX-induced cardiotoxicity mouse	15mg/kg, 30 mg/kg, 12 days	caspase-3↓, caspase-9↓, HAX-1↑, Bcl-2↑, LPS↓, IL-1β↓, TNF-α↓, IL-10↑, TGF-β↑, NF-κB↓,	Improvement of inflammation, Changes of	*Bacteroidetes/Firmicutes*↓, *Desulfovibrio*↓, *Lactobacillus*↑	Huang et ai.(2016)

						signal transducer and activator of transcription 6 (STAT6)↑	gut microbiota		
	Isoliquiritigenin	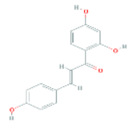	Isoliquiritigenin	High-fat diet mouse, FMT	2 weeks	glucose↓, TNF-α↓, monocyte chemoattractant protein-1 (MCP-1)↓, F4/80↓, CD11c↓	Improvement of inflammation and fat metabolism	*Bacteroides spp*↓, *Lactobacillus johnsonii*↓, *Lactococcus lactis*↓, *Firmicutes*↓	Ishibashi et al.(2022)
Rhizoma Alismatis	Alisol A 24-acetate	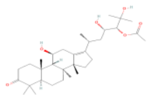	Alisol A 24-acetate	In vitro	10–50 µM, 2 days	HSL↓, adipose triglyceride lipase (ATGL)↑, PPARγ↓, perilipin A↓	Improvement of fat metabolism	-	Lou et al.(2021)
Crataegus pinnatifida Bung	Procyanidins	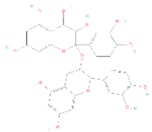	Procyanidins	Lipid metabolism disorder mouse	50mg/kg, 100mg/kg, 200 mg/kg, 8 weeks	T-CHO↓, TG↓, LD↓L, HDL↓, SOD↑, MDA↓, glutathione (GSH)↑, ALT↓, AST↓, insulin↓, LPS↓, adiponectin (ADPN), leptin, TLR4↓, NF-κB↓, MYD88↓,	Improvement of inflammation	*Akkermansia*↓, *Bacteroides*↓, *Bifidobacterium*↑, *Blautia*↑, *Lachnospiraceae*↑, *Subdoligranulum*↑	Han et al.(2022)

					IKKβ↓, TNF-α↓, IL-1β↓, AMPK↑, Acc↑			
		Procyanidins	Gestational diabetes mellitus mouse, feeding a high-fat-high-sucrose diet	27.8 mg/kg, 4 weeks	IL-6↓, TNF-α↓, IL-17↓, NF-κB↓, NLRP3↓	Improvement of inflammation	-	Liu et al.(2022)
Quercetin	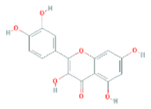	Combination of quercetin and resveratrol	High-fat diet mouse	30mg/kg, 10 weeks	IL-6↓, TNF-α↓, MCP-1↓	Improvement of inflammation, Changes of gut microbiota	*Bacteroidetes/Firmicutes*↑, *Lachnoclostridium*↓, *Ruminococcaceae_UCG-014*↑, *Ruminococcaceae_UCG-005*↑	Zhao et al.(2017)
Naringenin	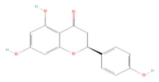	Naringenin	High-fat diet rats	100mg/kg, 12 weeks	WAT↓, O2 consumption↑, CO2 production↑, energy	Changes in energy and fat metabolism,	*Akkermansia*↑	Zhang et al.(2022)

						expenditure↑, beige adipose browning related-markers (CD137, HOXC8 and TBX1)↑, mitochondrion related markers (TFAM, NRF1/2, UCP1, PGC1α and TFAM)↑, acetic acid↑	Changes of gut microbiota		
Pericarpium Citri Reticulatae	Polymetho xyflavones<br><br>Hydroxyl polymetho xyflavones	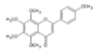<br><br>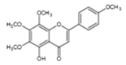	Polymethox yflavones and hydroxyl polymethox yflavones	High-fat diet mouse, in vitro	25μg/mL, 50μg/mL, 2 days	SREBP-1↓, perilipin 1↓, lipid↓	Changes in fat metabolism, Changes of gut microbiota	*Prevotella*↓, *rc4-4*↓	Tung et al.(2018)
	Hesperidin	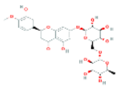	Hesperidin	High-fat diet mouse	0.2% wt/wt in diet, 16 weeks	TG↓, T-CHO↓, LDL↓, AST↓, ALT↓, fatty acid oxidation gene (PPARα↑, CPT1α↑, ACOX1↑, ACADM↑,	Changes in fat metabolism and inflammation , Changes of gut microbiota	*Bacteroidetes*/*Firmicutes*↑,*Bacteroides*↑, *Prevotellaceae*↑, *Bacteroides_sartorii*↑	Li et al.(2022)

					HADH↓, SREBP-1C↓,SCD1↓)			
Tangeretin	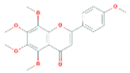	Tangeretin	High-fat diet mouse	100 mg/kg, 12 weeks	ALT↓, AST↓, T-CHO↓, TG↓, TNF-α↓, IL-6↓, LPS↓, Nrf2↑, NF-κB↓, IκB-α↓	Changes in fat metabolism, Changes of gut microbiota	*Bacteroidetes/Firmicutes*↑, *Bacteroides*↑, *Lactobacillus*↑	Chen et al.(2022)

Appendix 1: Formulas summary table

Formulas	TCM Drug Name	Full Botanical Name	Family	Part Used	Processing Method	Quantity of Herbs
Sijunzi Decoction	Ren Shen (ginseng)	*Panax ginseng* C.A.Mey.	Araliaceae	Root	Raw Dried	9 g
	Fu Ling (Poria cocos)	*Poria cocos* (Schw.) Wolf	Polyporaceae	Sclerotium	Raw Dried	9 g
	Bai Zhu (Atractylodes macrocephala Koidz)	*Atractylodes macrocephala* Koidz.	Asteraceae	Root	Raw Dried	9 g
	Gan Cao (Glycyrrhiza)	*Glycyrrhiza glabra* L.	Leguminosae	Root and rhizome	Honey-prepared	6 g
Chaishao Liujunzi Decoction	Chen Pi (Pericarpium Citri Reticulatae)	*Citrus reticulata* Blanco	Rutaceae	Pericarp	Raw Dried	10 g
	Ban Xia (Pinellia ternata)	*Pinellia ternata*（Thunb.）Breit.	Araceae	Rhizome	Ginger-processed	9 g

	Dang Shen	*Codonopsis pilosula* (Franch.) Nannf.	Campanulaceae	Root	Raw Dried	9 g
	Fu Ling (Poria cocos)	*Poria cocos* (Schw.) Wolf	Polyporaceae	Sclerotium	Raw Dried	9 g
	Bai Zhu (Atractylodes macrocephala Koidz)	*Atractylodes macrocephala* Koidz.	Asteraceae	Root	Raw Dried	9 g
	Gan Cao (Glycyrrhiza)	*Glycyrrhiza glabra* L.	Leguminosae	Root and rhizome	Honey-prepared	6 g
	Chai Hu	*Bupleurum chinense* DC.	Apiaceae	Root and rhizome	Raw Dried	10 g
	Bai Shao	*Paeonia lactiflora* Pall.	Ranunculaceae Juss.	Root	Raw Dried	10 g
Xiangsha Liujunzi Decoction	Sha Ren	*Amomum villosum* Lour.	Zingiberaceae	Ripened fruit	Raw Dried	4.8 g
	Mu Xiang	*Aucklandia lappa* Decne.	Asteraceae Dumort.	Root	Raw Dried	4.2 g

	Chen Pi (Pericarpium Citri Reticulatae)	*Citrus reticulata* Blanco	Rutaceae	Pericarp	Raw Dried	10 g
	Ban Xia (Pinellia ternata)	*Pinellia ternata*（Thunb.）Breit.	Araceae	Rhizome	Ginger-processed	6 g
	Dang Shen	*Codonopsis pilosula* (Franch.) Nannf.	Campanulaceae	Root	Raw Dried	9 g
	Fu Ling (Poria cocos)	*Poria cocos* (Schw.) Wolf	Polyporaceae	Sclerotium	Raw Dried	9 g
	Bai Zhu (Atractylodes macrocephala Koidz)	*Atractylodes macrocephala* Koidz.	Asteraceae	Root	Raw Dried	12 g
	Gan Cao (Glycyrrhiza)	*Glycyrrhiza glabra* L.	Leguminosae	Root and rhizome	Honey-prepared	6 g
Shenling Baizhu Powder	Ren Shen (ginseng)	*Panax ginseng* C.A.Mey.	Araliaceae	Root	Raw Dried	10 g
	Fu Ling (Poria cocos)	*Poria cocos* (Schw.) Wolf	Polyporaceae	Sclerotium	Raw Dried	10 g

	Bai Zhu (Atractylodes macrocephala Koidz)	*Atractylodes macrocephala* Koidz.	Asteraceae	Root	Raw Dried	10 g
	Shan Yao (Chinese yam)	*Dioscorea opposita* Thunb.	Dioscoreaceae	Root and rhizome	Raw Dried	10 g
	Bai Biandou	*Dolichos lablab* L.	Fabaceae	Mature seed	Raw Dried	7.5 g
	Lian Zi	*Nelumbo nucifera* Gaertn.	Nymphaeaceae	Mature seed	Raw Dried	5 g
	Yi Yiren	*Coix lacryma-jobi* L. var. *mayuen*（Roman.）Stapf	Poaceae Barnhart	Mature seed	Raw Dried	5 g
	Sha Ren	*Amomum villosum* Lour.	Zingiberaceae	Ripened fruit	Raw Dried	5 g
	Jie Geng	*Platycodon grandiflorum*（Jacq.）A. DC.	Campanulaceae Juss.	Root	Raw Dried	5 g
	Gan Cao (Glycyrrhiza)	*Glycyrrhiza glabra* L.	Leguminosae	Root and rhizome	Honey-prepared	10 g
Erchen Decoction	Chen Pi (Pericarpium Citri Reticulatae)	*Citrus reticulata* Blanco	Rutaceae	Pericarp	Raw Dried	15 g

	Ban Xia (Pinellia ternata)	*Pinellia ternata*（Thunb.）Breit.	Araceae	Rhizome	Ginger-processed	15 g
	Fu Ling (Poria cocos)	*Poria cocos* (Schw.) Wolf	Polyporaceae	Sclerotium	Raw Dried	9 g
	Gan Cao (Glycyrrhiza)	*Glycyrrhiza glabra* L.	Leguminosae	Root and rhizome	Honey-prepared	4.5 g
Daotan Decoction	Cang Zhu	*Atractylodes lancea*（Thunb.）DC.	Asteraceae Dumort.	Root and rhizome	Raw Dried	10 g
	Xiang Fu	*Cyperus rotundus* L.	Cyperaceae Juss.	Root and rhizome	Raw Dried	10 g
	Zhi Ke	*Citrus aurantium* L.	Rutaceae Juss.	Immature fruit	Raw Dried	10 g
	Ban Xia (Pinellia ternata)	*Pinellia ternata*（Thunb.）Breit.	Araceae	Rhizome	Ginger-processed	10 g
	Chen Pi (Pericarpium Citri Reticulatae)	*Citrus reticulata* Blanco	Rutaceae	Pericarp	Raw Dried	6 g

	Fu Ling (Poria cocos)	*Poria cocos* (Schw.) Wolf	Polyporaceae	Sclerotium	Raw Dried	15 g
	Tian Nanxing	*Arisaema erubescens*（Wall.）Schott	Araceae Juss.	Root and rhizome	Raw Dried	6 g
	Gan Cao (Glycyrrhiza)	*Glycyrrhiza glabra* L.	Leguminosae	Root and rhizome	Honey-prepared	6 g
	Gan Jiang	*Zingiber officinale* Rosc.	Zingiberaceae	Root and rhizome	Raw Dried	6 g
Huanglian Wendan Decoction	Ban Xia (Pinellia ternata)	*Pinellia ternata*（Thunb.）Breit.	Araceae	Rhizome	Ginger-processed	12 g
	Chen Pi (Pericarpium Citri Reticulatae)	*Citrus reticulata* Blanco	Rutaceae	Pericarp	Raw Dried	18 g
	Huang Lian	*Coptis chinensis* Franch.	Ranunculaceae Juss.	Root	Raw Dried	12 g
	Zhu Ru	*Bambusa tuldoides* Munro	Poaceae Barnhart	Rhizome	Raw Dried	12 g
	Zhi Shi	*Citrus aurantium* L.	Rutaceae Juss.	Young fruit	Raw Dried	12 g

Fu Ling (Poria cocos)	*Poria cocos* (Schw.) Wolf	Polyporaceae	Sclerotium	Raw Dried	9 g
Gan Cao (Glycyrrhiza)	*Glycyrrhiza glabra* L.	Leguminosae	Root and rhizome	Honey-prepared	6 g
Sheng Jiang	*Zingiber officinale* Rosc.	Zingiberaceae	Root and rhizome	Fresh	6 g

Glossary

**TCM**	traditional Chinese medicine	**ALT**	alanine aminotransferase
**BMI**	body mass index	**FAS**	fatty acid synthase
**WHO**	World Health Organization	**Acc**	acetyl-CoA carboxylas
**TGR5**	G protein-coupled bile acid receptor-5	**BAs**	bile acids
**TLR4**	toll-like receptor-4	**TβMCA**	tauro-β-muricholic acid
**LPS**	lipopolysaccharide	**GCA**	glycocholic acid
**SCFAs**	short-chain fatty acids	**GCDCA**	glycodeoxycholic acid
**GPR43**	G protein-coupled receptors 43	**GDCA**	glycerodeoxycholic acid
**GPR41**	G protein-coupled receptors 41	**TCA**	taurocholic acid
**TMAO**	trimethylamine-N-oxide	**TCDCA**	taurochenodeoxycholic acid
**PYY**	peptide YY	**TDCA**	taurodeoxycholic acid
**GLP-1**	glucagon-like peptide 1	**TαMCA**	tauro-α-muricholic acid
**TNF-α**	tumor necrosis factor-α	**TβMCA**	tauro-β-muricholic acid
**IL-6**	interleukin-6	**CA**	cholic acid
**IL-10**	interleukin-10	**CDCA**	chenodeoxycholic acid
**IL-8**	interleukin-8	**DCA**	deoxycholic acid
**IL-1β**	interleukin-1β	**βMCA**	β-muricholic acid
**sPLA2-X**	Group X phospholipase A2	**7-ketoLCA**	7-ketolithocholic acid
**HDAC**	histone deacetylase	**βCA**	β-cholic acid
**GALT**	gut-associated lymphoreticular tissue	**GF**	germ-free
**LPS**	lipopolysaccharide	**Mcad**	medium-chain acyl-CoA dehydrogenase
**EVs**	extracellular vesicles	**Mtp**	microsomal triglyceride transport protein
**AST**	aspartate transaminase	**LCA**	lithocholic acid
**CYP7A1**	cholesterol 7α-hydroxylase	**FXR**	farnesoid X receptor
**AMPK**	Adenosine 5‘-monophosphate -activated protein kinase	**AMP**	adenosine monophosphate
**ATP**	adenosine triphosphate	**ADP**	adenosine diphosphate

**GABA**	γ-amino butyric acid	**Glu**	glutamate
**Lac**	lactate	**GIP**	gastric inhibitory polypeptide
**GBA**	gut-brain axis	**Rap1**	rasrelatedprotein 1
**GAS**	gastrin	**MTL**	motilin
**SS**	somatostatin	**GI**	gastrointestinal
**GRHL**	Ghrelin	**IL-2**	interleukin-2
**IFN-γ**	interferon-gamma	**ROS**	reactive oxygen species
**MMP**	mitochondrial membrane potential	**LC3**	microtubule-associated protein light chain 3
**p62**	sequestosome 1	**ULK1**	unc-51 like autophagy activating kinase 1
**PINK1**	PTEN induced kinase 1	**mtROS**	mitochondrial reactive oxygen species
**PAX4**	paired box gene 4	**5-HT**	5-Hydroxytryptamine
**TPH1**	tryptophan hydroxylase-1	**5-HT3r**	5-hydroxytryptamine 3 receptor
**BA**	bile acid	**iNOS**	inducible nitric oxide synthase
**NO**	nitric oxide	**TLRs**	toll-like receptors
**TLR2**	toll-like receptor-2	**NF-κB**	nuclear factor kappa-B
**TNF-α**	tumor necrosis factor-alpha	**MAPK**	mitogen-activated protein kinase
**MyD88**	Myeloid Differentiation Primary Response Gene 88	**IL-1β**	interleukin-1β
**IFN-β**	interferon-beta	**IL-17**	interleukin-17
**MDA**	malondialdehyde	**SOD**	superoxide dismutase
**HDL**	high-density lipoprotein cholesterol	**LDL**	low-density lipoprotein cholesterol
**TG**	triglyceride	**T-CHO**	total cholesterol
**SS**	somatostatin	**CCK**	concentrations of cholecystokinin
**IL-18**	interleukin-18	**NLRP3**	the NOD-like receptor family, pyrin domain containing 3 inflammasome
**Hb1Ac**	glycated hemoglobin	**LPL**	Lipoprotein lipase
**PPARγ**	peroxisome proliferator-activated receptor gamma	**IRS1**	insulin receptor substrate 1
**PKA**	protein kinase A	**HSL**	hormone-sensitive triglyceride lipase
**AKT**	protein kinase B	**ITT**	insulin tolerance test
**FFA**	free fatty acids	**HDAC1**	histone deacetylase 1

**H3K9ac**	histone H3 lysine 9 acetylation pathway	**GSH-Px**	glutathione peroxidase
**PCOS**	polycystic ovary syndrome	**T**	testosterone
**LH**	luteinizing hormone	**FSH**	stimulating growth hormone
**E2**	estradiol	**OATP3A1**	organic anion transporting polypeptides-3A1
**OATP2B1**	organic anion transporting polypeptides-2B1	**ISI**	insulin sensitivity index
**IRI**	insulin resistance index	**2hPG**	2-hour postprandial glucose
**IRS-1**	insulin receptor substrate 1	**BAT**	brown adipose tissue
**UCP1**	uncoupling protein 1	**OXPHOS**	oxidative phosphorylation
**AchE**	acetylcholinesterase	**SP**	substance P
**VIP**	vasoactive intestinal peptide	**T4**	thyroxine
**T3**	triiodothyronine	**CS**	citrate synthase
**NCR**	NADPH-cytochrome C reductase	**IDH1**	isocitrate dehydrogenase 1
**cGMP**	cyclic guanosine monophosphate	**ACTH**	adrenocorticotropic hormone
**cAMP**	cyclic adenosine monophosphate	**CORT**	cortisol
**IκB-α**	inhibitor of κB-alpha	**PGE2**	prostaglandin E2
**PGD2**	prostaglandin D2	**TXB2**	thromboxane B2
**HETE**	hydroxyeicosatetraenoic acids	**HODE**	hydroxyoctadecadienoic acids
**LXR**	liver X receptor	**MPO**	myeloperoxidase
**Nrf2**	nuclear respiratory factor 2	**NOX1**	NADPH oxidase 1
**DUOX2**	dual oxidase 2	**FPR**	formyl peptide receptor
**FGF**	fibroblast growth factor	**UDCA**	ursodeoxycholic acid
**IKKα**	nuclear factor kappa-B kinase subunit alpha	**ALP**	alkaline phosphatase
**CHE**	cholinesterase	**FASN**	fatty acid synthase
**MGL**	acylglycerol lipase	**TNF-β**	tumor necrosis factor-beta
**STAT6**	signal transducer and activator of transcription 6	**MCP-1**	monocyte chemoattractant protein-1
**WAT**	white adipose tissue	**ATGL**	adipose triglyceride lipase
**GSH**	glutathione	**ADPN**	adiponectin

**IKKβ**	nuclear factor kappa-B kinase subunit beta	**PMFs**	polymethoxyflavones
**HPMFs**	hydroxyl polymethoxyflavones		
